# Scarcity, cognition, and health decision-making: evidence from a lab-in-the-field experiment in rural China

**DOI:** 10.1038/s41598-026-71861-z

**Published:** 2026-10-28

**Authors:** Haiou Zhu, Fangzhou You, E. Liu, Thorsten Gruber, Hua Dong, Cees De Bont

**Affiliations:** 1 Department of Psychiatry, University of Oxford, Oxford, UK; 2 Centre for Doctoral Training in AI for Digital Media Inclusion, Royal Holloway, University of London, London, UK; 3 School of Arts and Creative Technologies, University of York, York, UK; 4 School of Economics and Management, Yunnan Minzu University, Kunming, China; 5 Centre for Service Management, Business School, Loughborough University, Loughborough, UK; 6 Helen Hamlyn Centre for Design, Royal College of Art, London, UK; 7 College of Design and Engineering, National University of Singapore, Singapore, Singapore

**Keywords:** Scarcity, Psychology of poverty, Cognitive function, Present bias, Risk aversion, Health decision-making, Health inequality, Environmental social sciences, Health care, Psychology, Psychology

## Abstract

Understanding how financial scarcity impacts health-related decision-making is vital for designing interventions to break the cycle between poverty and poor health. We conducted a lab-in-the-field experiment with 479 rural adults in two low-income regions of China, Yunnan and Shaanxi, to examine the cognitive and behavioural consequences for health-related preferences and choices. Participants were randomly assigned to an acute financial scarcity intervention consisting of three hypothetical scenarios featuring either a heavy financial burden (the Hard prime, e.g., raising 20,000 yuan within a week) or a minimal cost (the Easy control, 1,000 yuan). We measured cognitive function using a 12-item Raven’s Progressive Matrices task, time and risk preferences across monetary and health domains using multiple price lists and ordered lottery menus, and spending decisions using a 100-yuan budget allocation task. In the moderately poor region of Yunnan, the scarcity prime significantly reduced cognitive performance by 8.4% points and increased health-related risk aversion, driving individuals toward safer vaccine options. In contrast, no cognitive or behavioural preference shifts were observed in the more severely impoverished region of Shaanxi. Intertemporal time preferences remained stable across both regions and choice domains. Budget allocations showed limited overall shifts, though specific resource-scarce subgroups reallocated spending more toward health items. These findings demonstrate that the cognitive and behavioural consequences of scarcity are not universal but highly context-dependent, shifting with the chronic depth of underlying deprivation.

**Supplementary Information:**

The online version contains supplementary material available at https://doi.org/10.1038/s41598-026-71861-z.

## Introduction

 Health-related decision-making often requires individuals to plan for the future and invest resources in preventive or long-term benefits^1,2^. However, the reality of scarcity, having insufficient resources for one’s needs, can undermine such forward-looking health behaviours^3,4^. Households facing tight budgets tend to prioritise immediate necessities like food or shelter over expenditures such as medications or preventive services (annual check-ups, immunisations)^5^. Living in poverty has also been linked to certain unhealthy behaviours, for example choosing cheaper, calorie-dense foods or spending on “temptation goods” (like alcohol or cigarettes) despite long-term health costs^6^. Indeed, poverty contributes to a vicious cycle in which poor health outcomes both result from and exacerbate economic hardship in low-income communities^7^. This is evident in rural China, as in many rural areas globally, that illness is a leading cause of poverty^8^. Rural regions face stark disparities in healthcare access compared to cities, and vulnerable groups such as the elderly, women, and left-behind children (those with migrant worker parents) suffer disproportionately from unmet health needs^9,10^. Understanding the relationship and underlying mechanisms between health decision-making and poverty is essential for designing interventions to break these traps and reduce health inequities^11,12^.

Scarcity theory in economics provides an integrative framework for how poverty impacts decision-making^13,14^. Classical economics traditionally focuses on objective scarcity, defined as an absolute shortfall in material resources, and how it constrains consumption and investment^2^. By contrast, behavioural and public economics increasingly highlight the nuanced, subjective nature of resource strain. Within this paradigm, scarcity is conceptualised psychologically as ‘having less than you feel you need’^14^. The psychology of poverty describes a scarcity mindset, where both monetary concerns (e.g. paying for rent, school fees, utility bills) and non-monetary hardships (poor healthcare access, malnutrition, lack of sleep) preoccupy one’s thoughts, making everyday decisions more difficult and stressful^15,16^. This perspective emphasises that poverty entails psychological and cognitive scarcity as much as material deprivation^15^. Even among higher-income individuals, acute worries, such as during an economic crisis, a pandemic lockdown, or concerning climate change, may impair cognitive performance and decision-making^17,18^. The key idea of scarcity theory is that scarcity itself induces a distinctive mindset that influences how people think and decide, and subsequently affect human preferences and behaviours^13^.

Two core psychological mechanisms have been proposed to explain decisions under scarcity: cognitive load and tunneling^13,19,20^. First, poverty-related concerns can occupy a portion of one’s mental bandwidth, leaving fewer cognitive resources available for other tasks^21,22^. While some experiments show that financial worries directly impair cognitive performance^21,22^, others find no significant cognitive decline under scarcity conditions^23–25^. A recent meta-analysis found a moderate negative effect of scarcity on cognitive function, though effect sizes varied with context and individual differences (e.g., education level, severity, and timing of scarcity)^26^. Second, feeling resource-constrained can lead to tunnelling, a narrowing of focus and attention^15,27^. Some evidence suggests that scarcity-induced tunnelling may improve decision-making by eliminating distractions^28^, increase consistency in how people value products^29^, or even increase cognitive performance when paired with incentives^30^. On the other hand, studies have linked tunneling to counter-productive choices such as over-borrowing^31^, impulse purchase^32^, and poor budgeting (failing to plan expenditures)^33^. Whether scarcity leads to more efficient decisions or worse ones likely depends on which psychological mechanism (taxing cognitive load vs. enhancing focus) dominates in a given context.

Scarcity may also influence time and risk preferences, which in turn drive various behaviours^16,19^. Time preference refers to the way individuals make decisions over time, trading off present and future outcomes, and numerous studies have observed that people tend to weight near-term outcomes more heavily than distant ones^34^. Present bias captures this tendency as a quasi-hyperbolic discount function, in which a person prefers an immediate trade-off to the same trade-off shifted into the future^35^. Risk preference describes decision-making under uncertainty when people weigh the probabilities of potential outcomes, while risk aversion describes the tendency to prefer a certain outcome over a risky prospect of equal or higher expected value^36^. Scarcity might intuitively be expected to exacerbate present bias and risk aversion, as those under financial strain prioritise immediate needs and security^37,38^. Indeed, some studies report that low-income households appeared more present-biased in monetary choices shortly before payday than after^39^ and poorer villagers were more risk averse^37^. However, other field studies find that short-term income fluctuations leave measured time preferences largely unchanged^25,40^ or even reduced risk aversion^24^. A recent systematic review of 67 rural time-preference studies concludes that much of the apparent variation in measured time preferences reflects differences in how preferences are conceptualised and measured rather than scarcity itself^41^. These mixed results indicate that scarcity does not uniformly heighten present bias or risk aversion^13^.

Another possible explanation for the mixed findings on how scarcity affects decision-making is that the effects may differ depending on the decision domain involved^42–44^. People often behave differently when the consequences involve health rather than money. For example, individuals tend to be more risk-averse for outcomes that affect health (such as choosing medical treatments) than for financially framed outcomes^45^. Likewise, individuals sometimes display a stronger present bias for health benefits compared to monetary gains, perhaps because immediate health improvements are valued particularly strongly^46^. These domain differences suggest the need to explicitly study health decisions under scarcity, rather than assume findings from financial decisions can be generalised. However, existing research on scarcity has predominantly emphasised financial decision-making^13,19,47^, or explored scarcity’s impacts on behaviours related to productivity^48^, parenting and investment in children’s education^49,50^ and savings^51^. There is a lack of research examining how scarcity influences health-related decisions, despite the central role that health decisions play in quality of life and long-term economic outcomes.

Drawing on scarcity theory and given the mixed evidence on mechanisms and their consequences, the present study aimed to examine the effects of financial scarcity on (i) cognitive function, (ii) time and risk preferences in both monetary and health domains, (iii) spending trade-off decisions, among low-income adults in rural China. We implemented a lab-in-the-field experiment in two distinct rural regions, Yunnan and Shaanxi provinces of China. We randomly assigned participants to either an urgent high-cost expenses scenario (“Hard” scarcity prime; for example, needing to raise ¥20,000 within one week for an unexpected expense) or an otherwise identical low-cost scenario (the “Easy” control; the same event costing only ¥1,000), following methods used by prior studies^21,24,25^. Rural China is a particularly informative setting for this question. Although China declared the elimination of absolute poverty in 2021, illness-driven and relative poverty persist, making forward-looking health decisions under financial pressure a pressing policy concern^52^. Methodologically, it combines pronounced within-country heterogeneity in the depth of poverty with a shared language, currency, and policy environment, so that scarcity effects can be compared across regions differing in deprivation. Both regions are low-income and predominantly agricultural, but they differ in important socio-economic aspects (see details in Results). This two-site design allowed us to test whether scarcity effects are general or context-specific.

We also adopted a multi-dimensional perspective on scarcity, examining three different sources of scarcity: (1) immediate financial worry (induced scarcity via a financial priming task), (2) objective scarcity (household annual income), and (3) liquidity fluctuation (the number of days since participants’ last payment). These factors have each been identified in prior work as important predictors of subjective or objective scarcity, but most previous studies examine them in isolation^22,53^. By considering them jointly, we can explore whether multiple scarcity pressures interact or moderate each other’s effects. In terms of outcomes, we measured cognitive functioning using the 12-item Standard Progressive Matrices (a Raven’s matrices test)^54^. We measured time preference using multiple price list (MPL), intertemporal-choice tasks framed once as a cash payment and once as a health subsidy^35^, and risk preference using Eckel–Grossman tasks framed once as monetary lotteries and once as vaccine options^55^. Finally, we included a ¥100 budget allocation task to observe how participants prioritise spending between groceries (basic needs), health, and temptation goods when faced with a constrained budget. Relative to recent work on context-dependent and mixed scarcity effects, our intended contribution is twofold: (a) to test the identical scarcity prime across two regions of differing deprivation, isolating context-dependence from design differences; and (b) to extend scarcity measurement from the financial to the health domain through health-framed risk and time-preference tasks and a budget task with health-related choices, allowing domain-specific effects to surface rather than assuming that findings from financial decisions generalise to health.

## Results

### Sample characteristics and balance check

A sample of 479 adults from rural Yunnan (*N* = 264) and Shaanxi (*N* = 215) was included in the main analysis (see data exclusion details in “Methods”). Summary demographics and a balance check for the random assignment are provided in Supplementary Table S1. In Yunnan, 53.0% of participants were male, compared to 39.5% in Shaanxi. Over half of all participants (53.8% in Yunnan; 66.1% in Shaanxi) were aged 50 or above. A notable proportion (18.6% in Yunnan; 13.5% in Shaanxi) self-reported as illiterate. Annual household incomes differed starkly between the regions, reflecting different levels of scarcity: Yunnan participants reported a mean income of ¥28,462 ($4,233 USD), more than double the Shaanxi sample’s mean of ¥11,122 ($1,654 USD). Additionally, about half of the participants relied on small-scale farming or migrant labour as their primary income source (48.9% in Yunnan; 64.7% in Shaanxi). In Shaanxi, the majority (71.6%) reported having no fixed or regular income, versus 44.3% in Yunnan. Correspondingly, the time since last payday was much longer on average in Shaanxi (mean = 95 days) than in Yunnan (mean = 31 days), indicating more irregular cash flows in the Shaanxi communities. Despite these between-region differences, the randomisation to Hard vs. Easy prime conditions was generally successful within each region. The groups assigned to the Hard vs. Easy scenario did not significantly differ on most background characteristics. Two modest imbalances were noted in Shaanxi: participants in the Easy group had slightly higher education levels on average than those in the Hard group (*P* = 0.002), and a greater proportion of the Easy group had a recent physical health exam (*P* = 0.04).

### Manipulation check

**Fig. 1:**
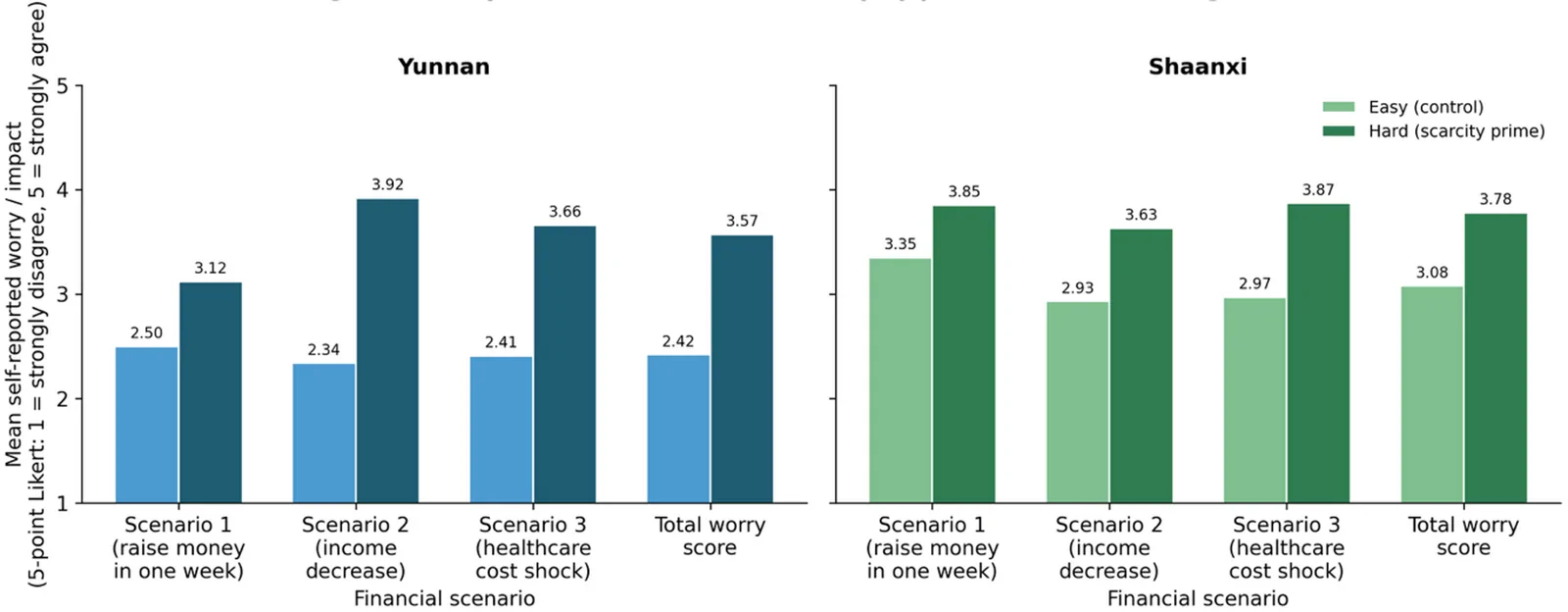
Grouped bar chart of mean self-reported worry/stress (1–5) for three financial scenarios, Hard vs. Easy, in Yunnan and Shaanxi; Hard bars are substantially higher in every scenario and region (all P < 0.001), with 95% CI error bars

The scarcity prime was effective in inducing greater subjective financial concern in the Hard condition compared to the Easy condition in both regions (Fig. 1). As intended, participants presented with the Hard scenarios reported substantially higher levels of worry and stress than those given the Easy versions. This pattern held true for all three vignettes and in both regions. Across all scenarios, the differences between Hard and Easy groups are statistically significant (*P* < 0.001 by t-tests; see SI Table S2 for the exact stress ratings and statistics). Thus, our priming manipulation successfully created an acute sense of financial scarcity in Hard-condition participants relative to controls, which serves as a foundation for examining its causal effects on cognitive and choice outcomes.

### Cognitive function

**Fig. 2:**
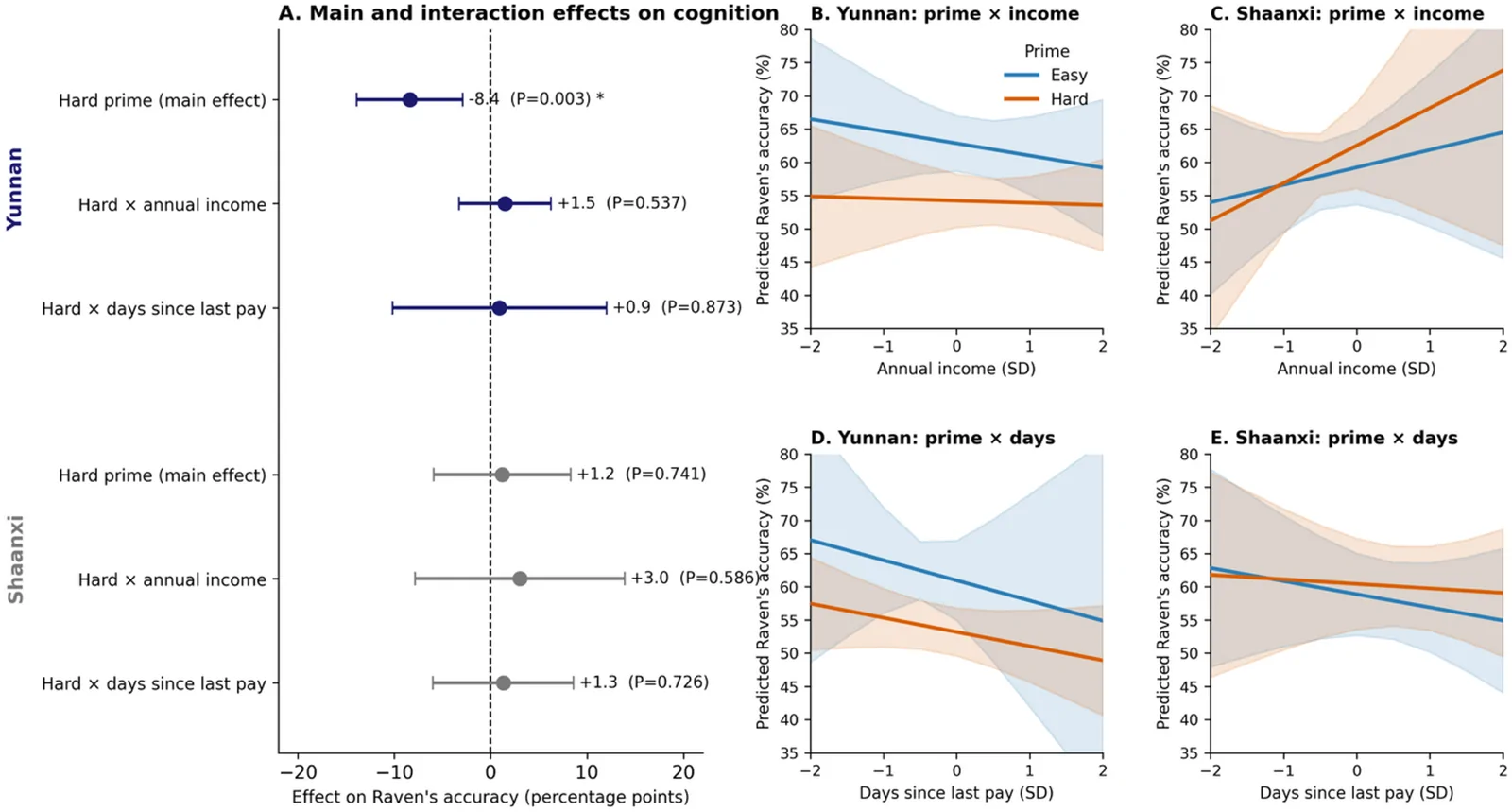
Forest plot (**A**) of the Hard-prime effect on Raven's accuracy and its interactions with income and days since last payment, by region, significantly negative in Yunnan, null in Shaanxi, no significant interactions; panels **B–E** plot predicted accuracy across each moderator for Easy vs. Hard.

Cognitive performance was measured by the accuracy rate on the 12-item Standard Progressive Matrices. Inducing a scarcity mindset impaired cognitive performance in Yunnan but not in Shaanxi. In Yunnan, the Easy group scored 61.5% on average on the 12-item Raven’s task, compared to 54.6% in the Hard group. Regression analysis (see SI Table S3) confirmed a significant negative effect of the Hard prime, an average drop of 8.4% points (*P* < 0.01). In contrast, Shaanxi participants showed no meaningful difference (60.2% Hard vs. 58.4% Easy). In other words, prompting thoughts of urgent financial needs reduced cognitive performance in Yunnan but had no effect in Shaanxi. We tested whether this effect varied by annual income or days since last payment (Fig. 2), but neither interaction was significant in either region (see SI Table S3).

### Time preference (present bias)

We examined whether scarcity influenced time preferences using multiple price list tasks for monetary and health choices. Present bias was measured as a quasi-hyperbolic pattern: switching from the sooner, smaller reward in the immediate (today vs. one month) list to the later, larger reward in the structurally identical delayed (six vs. seven months) list (see “Methods” section for detailed measurements). Only participants who made consistent choices, defined as switching their preference no more than once in each price list, were included in the analysis. In Yunnan, the Hard prime group was slightly less likely to show present bias in monetary choices (48.1%) than the Easy group (55.2%), though this difference was not statistically significant at conventional thresholds (*P* = 0.065; 95% CI for the prime coefficient − 1.07 to 0.03). No difference was found in health choices (40.2% vs. 39.5%). In Shaanxi, present bias rates were similar across conditions in both domains, with no significant effects (see SI Tables S4.1-4.2). As a robustness check that does not condition on consistency, we regressed the continuous discounting-ratio measure (β, defined for all 479 participants, including those classified as inconsistent) on the prime and the full covariate set, separately by region. The prime had no significant effect in any region or domain (monetary: Yunnan b = 0.009, *P* = 0.43; Shaanxi b = 0.013, *P* = 0.61; health: Yunnan b = − 0.014, *P* = 0.26; Shaanxi b = − 0.014, *P* = 0.38; SI Tables S5.1–S5.2), confirming that the null time-preference results are not an artefact of restricting the analysis to consistent decision-makers.

**Fig. 3:**
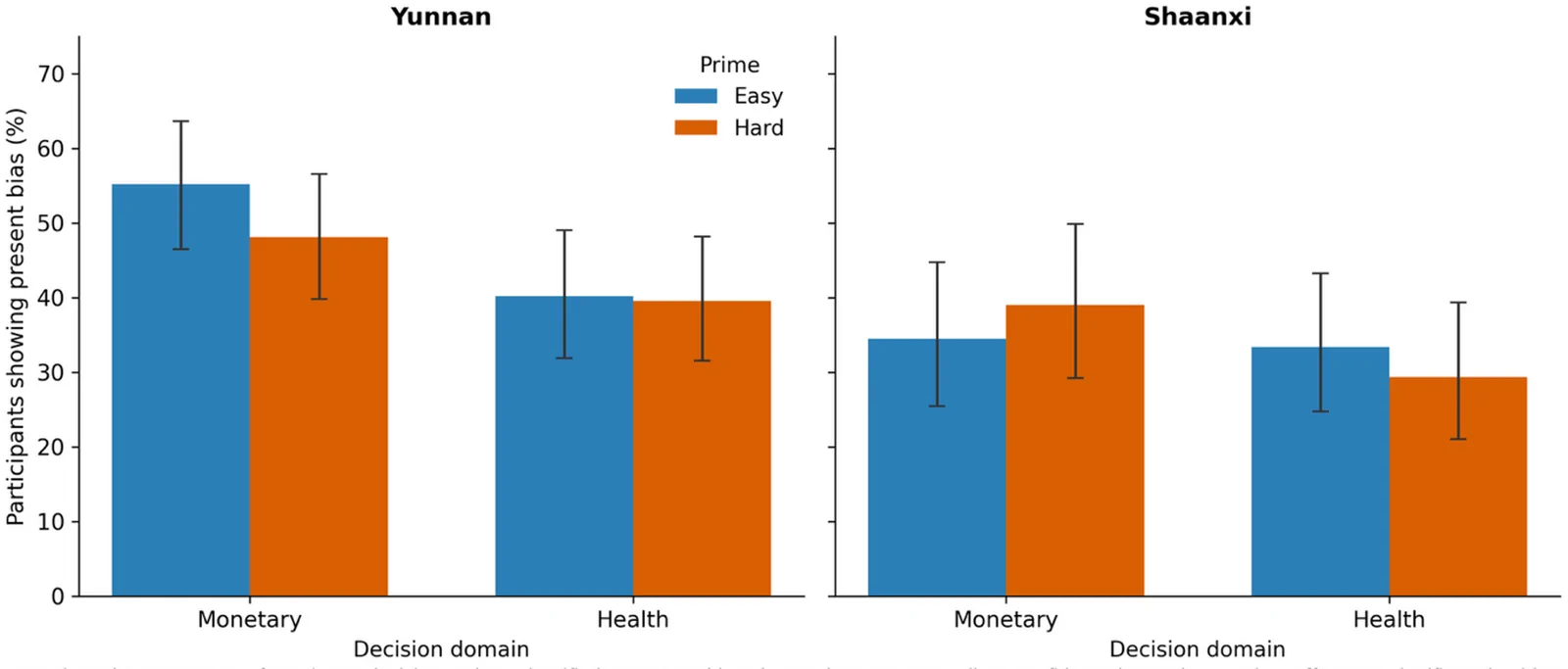
Grouped bars of the percentage of consistent decision-makers classified as present-biased, by domain, condition, and region; Hard–Easy differences small and non-significant throughout, with 95% Wilson CIs.

### Risk preference (risk aversion)

**Fig. 4:**
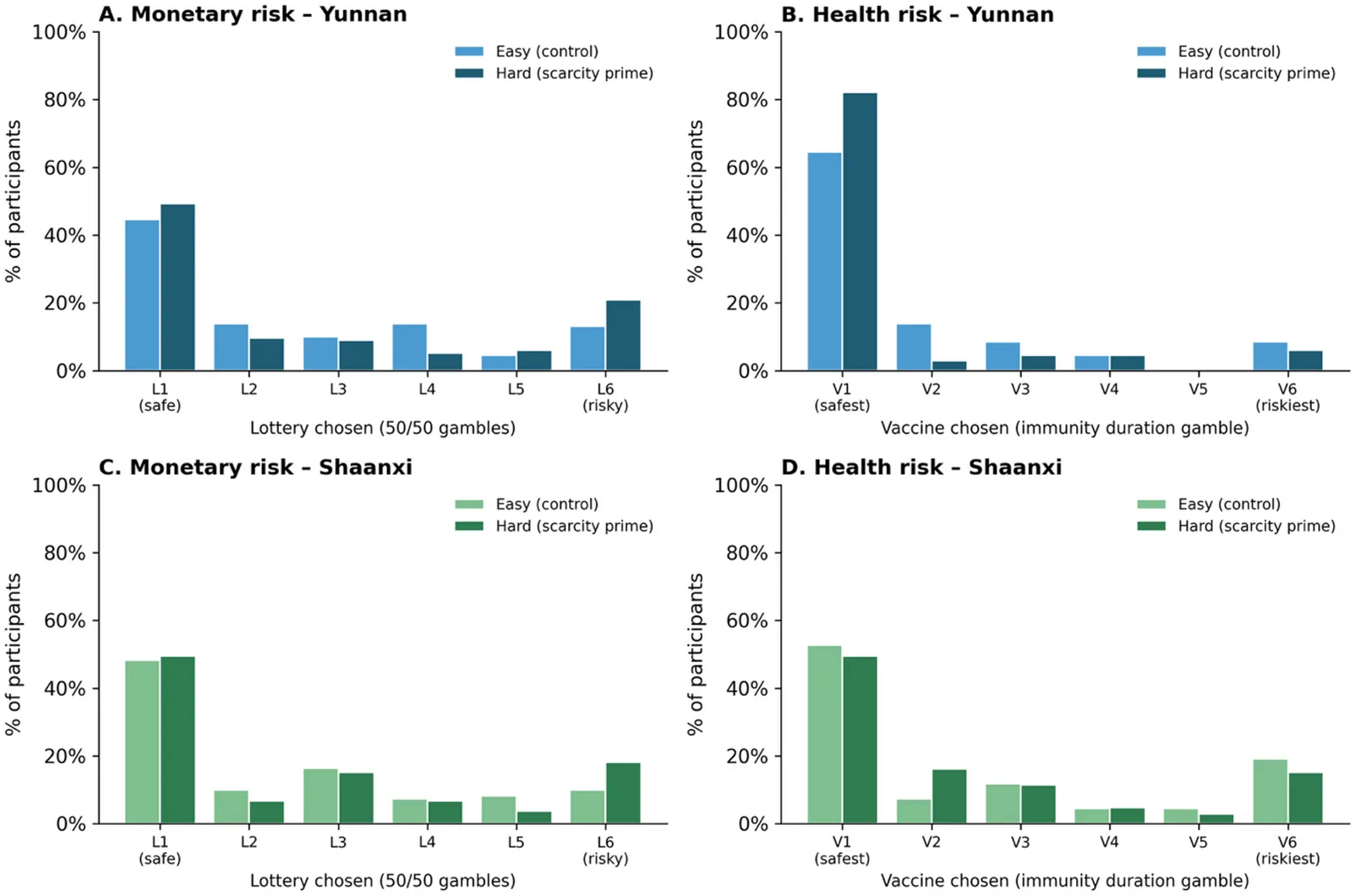
Four panels showing choice distributions across six options ordered low-to-high risk (monetary and health × two regions); the Hard prime shifts Yunnan health choices toward the safest options (**B**; ordered logit P = 0.008), with no effect for monetary risk or in Shaanxi.

We measured risk-taking behaviour through two parallel tasks: one involving a monetary lottery game and one involving vaccine options traded off health benefit against the probability of an adverse reaction (the safer option was the one with the lowest probability of side effects and a correspondingly modest benefit). Risk aversion was measured as choosing the more certain or safer options. For monetary risk, the Hard prime did not significantly change risk preference in either region (Fig. 4A and C). Ordered logistic regressions confirmed no meaningful prime effect on financial risk-taking (see SI Table S6). For health-related risk, participants under the Hard prime became significantly more risk averse in Yunnan sample (Fig. 4B). The Hard-primed individuals were more likely to opt for the safer vaccine option rather than a riskier option. In the Easy condition, 64.6% of Yunnan participants chose the safest vaccine, whereas under the Hard condition this percentage increased to 82.1%. An ordered logistic regression confirmed a significant effect of the prime (*P* = 0.008) after controlling for covariates. By contrast, no significant prime effect on health risk-taking was found in Shaanxi (Fig. 4D).

### Spending allocation decisions

A key question in our study was whether scarcity affects how people allocate spending across essential (groceries), health-related, and temptation goods. Across both samples, the largest budget share went to groceries, followed by health and temptation goods (see SI Table S4.1–4.2). We used seemingly unrelated regression (SUR) models to assess the effects of scarcity priming and its interaction with annual income and days since last pay (see SI Tables S7.1–S7.2). In Yunnan, there were no significant main effects of scarcity on any spending category. Income positively predicted health spending (β = 0.234, *P* = 0.025), but the interaction with scarcity was non-significant, as were all interactions with liquidity. In Shaanxi, scarcity priming also showed no main effects. However, interactions analysis shows that higher income group reduced grocery spending (Group × Income: β = − 0.63, *P* = 0.046) under the Hard prime. Interactions involving days since last payment were not significant in either region.

**Fig. 5:**
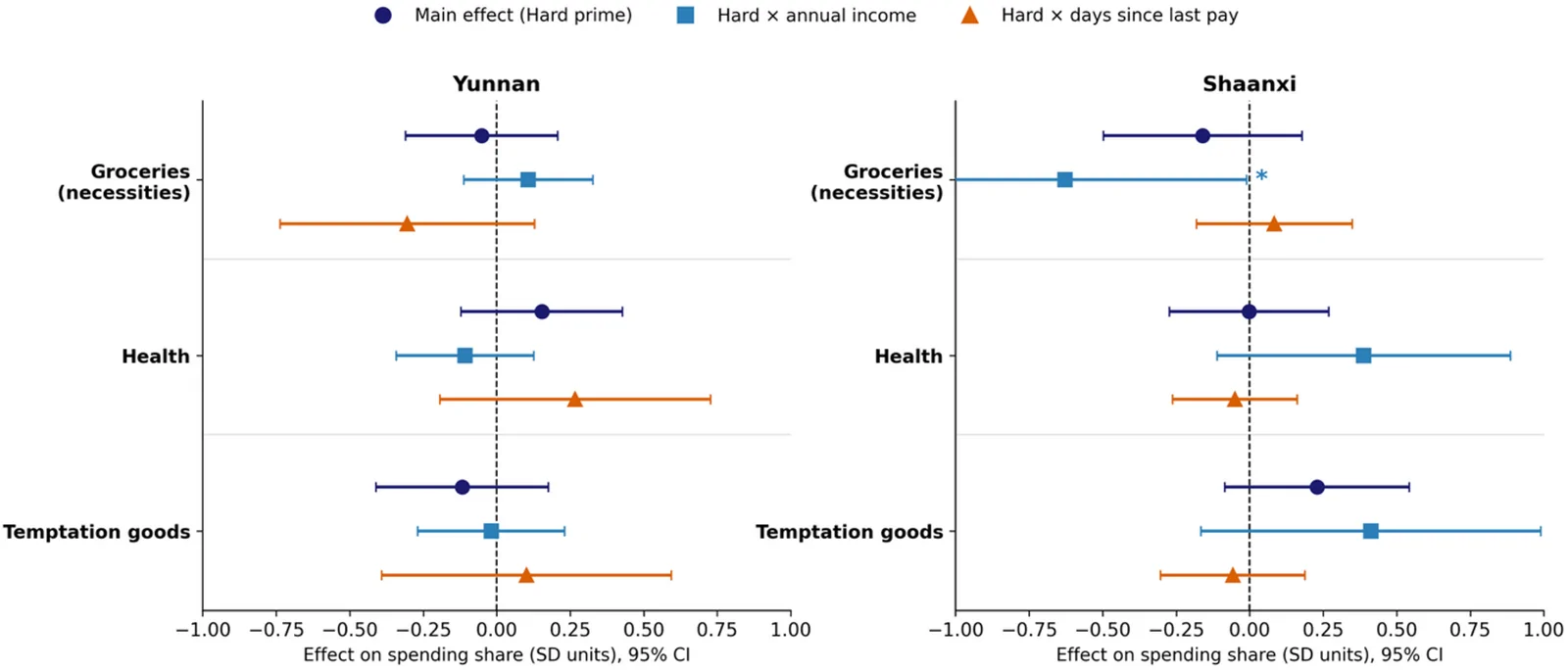
Coefficient plot of the prime's main effect and its income/liquidity interactions on standardised budget shares (groceries, health, temptation) by region, with 95% CIs; nearly all overlap zero, the only significant effect a negative Group × Income interaction for grocery spending in Shaanxi.

### Subgroup analysis

We ran subgroup analyses to explore heterogeneity in the effects of scarcity priming (Hard vs. Easy) on cognitive function and spending allocations across grocery, health, and temptation goods in Yunnan. We focused on Yunnan because the prime produced detectable average effects in this region, making it the natural setting in which to explore for whom those effects were largest. The parallel Shaanxi subgroup analyses, where no average effects were present, are reported in the Supplementary Information (Tables S9.1–S9.4). Each model included socio-demographic controls, excluding those overlapping with the subgroup variable. Exposure to the Hard condition significantly reduced cognitive scores across several subgroups, especially those with no income (β = − 25.58, *P* = 0.01), unfixed income (β = − 10.25, *P* = 0.03), lower income (β = − 9.64, *P* = 0.02), longer delay since last payment (β = − 12.35, *P* < 0.001), and smaller last payment (β = − 16.77, *P* < 0.001). Similar effects were found among women (β = − 10.7, *P* = 0.01), older adults (β = − 9.43, *P* = 0.02), and those without recent health exams (β = − 10.72, *P* = 0.05). In spending allocation, scarcity reduced grocery shares among participants with lower last payments (β = − 0.07, *P* = 0.04) and women (β = − 0.06, *P* = 0.09). Health spending increased under scarcity among those with lower income (β = 0.05, *P* = 0.09), longer delay since last pay (β = 0.09, *P* = 0.02), lower last payment (β = 0.07, *P* = 0.03), and women (β = 0.06, *P* = 0.08). Temptation spending remained largely unchanged, except for a decline among lower-income participants (β = − 0.03, *P* = 0.09).

**Fig. 6:**
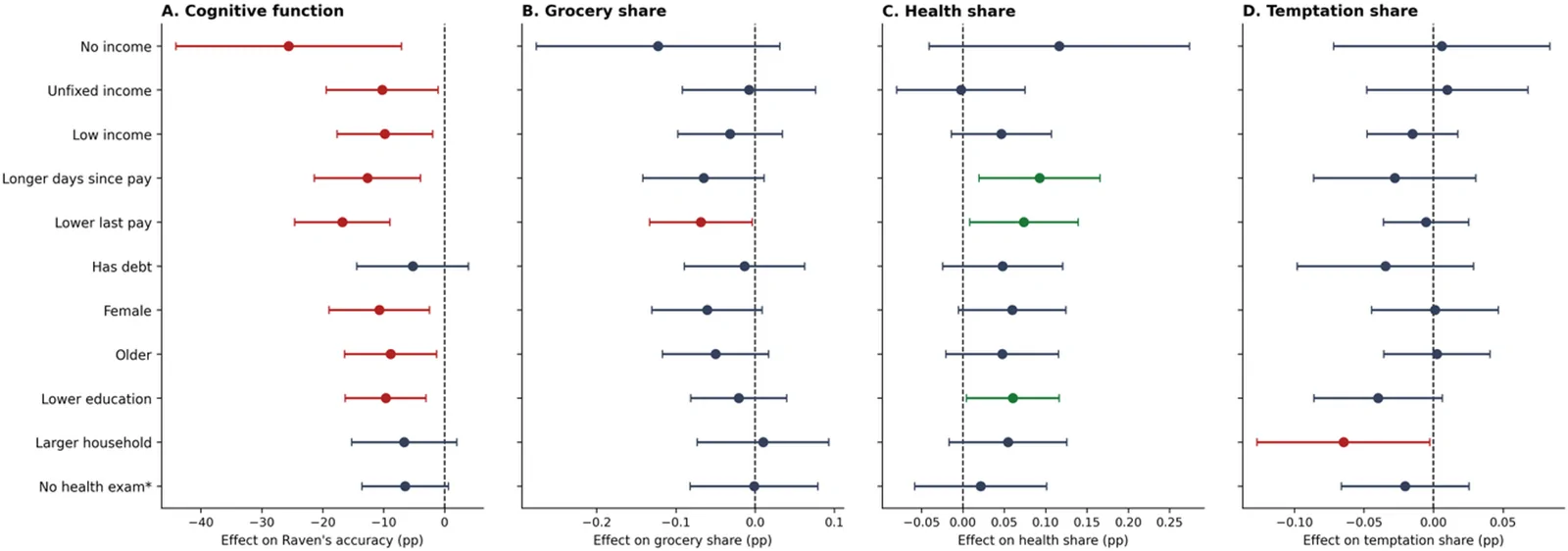
Coefficient plot of the Hard-prime effect within subgroups in Yunnan; panel **A** cognition (negative = worse under scarcity), **B–D** budget-share proportions; significant negative in red, positive in green, largest cognitive deficits among the most resource-scarce subgroups.

## Discussion

This study examined how scarcity affects cognitive functioning, time and risk preferences, and budget allocations across economic and health domains by comparing two low-income populations facing different depths of deprivation. We investigated three facets of scarcity: an immediate financial worry, chronic income level, and liquidity fluctuation (days since last payment). The core finding reveals that the psychological and behavioural consequences of scarcity are highly context-dependent rather than universal. Specifically, inducing acute financial worries via an experimental prime significantly impaired fluid intelligence and increased health-framed risk aversion in the moderately poor region of Yunnan, whereas no such cognitive or behavioural shifts occurred in the more severely impoverished region of Shaanxi. Meanwhile, time preferences remained largely stable across both regions and choice domains under acute scarcity priming.

One of the salient results was the cognitive tax of scarcity observed in Yunnan but not in Shaanxi. Participants from Yunnan who were primed with hard financial scenarios performed significantly worse on the Raven’s matrices, consistent with the idea that even momentary financial anxiety can sap mental bandwidth. This aligns with the findings that worrying about economic problems impairs cognitive capacity^21,22^. At the same time, the absence of any cognitive decline in the Shaanxi sample is equally noteworthy, especially since manipulation checks confirmed that the hard prime successfully generated elevated stress and worry in both regions. Several possible explanations merit consideration. One possibility is a ceiling or adaptation effect that Shaanxi participants, being substantially poorer, may be so habituated to financial hardship that an additional hypothetical challenge does not measurably alter their thinking. Second, a cognitive-floor effect is possible, whereby chronic deprivation has already depressed available bandwidth so far that the prime cannot lower it further. Finally, the regions may differ in task comprehension, as lower literacy in Shaanxi could introduce noise that obscures a true effect. Our findings are consistent with meta-analytic evidence which finds cognitive effect at a moderate level overall but shows it falling sharply once education is taken into account and growing with the severity of deprivation^26^. Our Shaanxi sample was both less educated and more severely deprived than our Yunnan sample, the combination under which an additional hypothetical prime is less likely to influence cognitive performance.

In terms of time preferences, Yunnan participants in the Hard condition showed a small, non-significant reduction in present bias for monetary choices and no change in health choices. Shaanxi participants showed no change in time preference with the prime in either domain. Our findings align with broader evidence that time preferences remain stable once measurement variations are controlled^41^. With respect to risk preferences, we found a domain-specific effect that immediate financial worries made Yunnan participants more conservative in health choices but not in monetary choices. This is consistent with prior observations that people are generally more risk-averse for health than money, and our results indicate scarcity amplifies that tendency, at least in the Yunnan context^46^. That this effect appeared only in Yunnan and only for health, while Shaanxi preferences were flat across conditions in both domains, reinforces the broader conclusion that scarcity’s effects on preferences are domain- and context-specific rather than uniform.

The budget al.location task provides insight into how acute scarcity shifts spending priorities among necessities (groceries), health, and temptation goods. In the full sample, the Hard prime did not produce statistically significant reallocations on average but the subgroup analyses in Yunnan sample revealed a more nuanced pattern. It was the participants with the scarcest real resources, for example, those with lower income or those who were farthest from their last payday, who showed the largest adjustments under the Hard prime. Generally, our results are consistent with scarcity theory that the poor often make consistent consumption decisions focusing on necessities^28^.

These context-dependent findings have profound implications for anti-poverty and public health intervention designs. In environments of moderate poverty like Yunnan, light-touch behavioural interventions and strategic targeting hold significant promise, especially in shifting attention and choices toward long-term health. Policymakers can alleviate cognitive loads by simplifying health logistics, automating basic medical insurance enrolment, or providing structural administrative assistance^56^. For example, simplifying the process of accessing healthcare (e.g. providing assistance with paperwork, sending reminders for check-ups, or using default enrolment in basic insurance programmes) can reduce the mental effort required to make healthy choices. Another strategy is behavioural targeting, which involves timing and tailoring interventions to moments and groups where they will have the greatest impact^57^. We found, for example, that women and those with irregular income (long gaps since last pay) experienced larger cognitive deficits and made bigger spending adjustments under scarcity. These groups might benefit from extra support, such as providing financial counselling, budgeting tools, or priority access to healthcare services^58^. In contrast, for populations trapped in hyper-chronic deprivation like Shaanxi, purely psychological nudges or priming adjustments are fundamentally insufficient to drive behavioural change. Instead, interventions must alter the actual choice infrastructure by blending behavioural techniques with robust structural safety nets. Because extremely poor households highly value long-term health but simply lack the material liquidity to afford it, implementing conditional cash transfers, health vouchers, or subsidised grant programmes targeted during lean seasons represents the most viable pathway to break the poverty-illness cycle^59^.

It is important to acknowledge the limitations of this study. First, while our sample covers two distinct regions, it is not representative of all rural populations. Caution is needed in generalising these results beyond the specific context of rural China. Since our two regions differed on multiple dimensions (age, income, culture), investigating which aspect was key could be done with a larger sample across more varied communities. Second, our outcome measures might simplify complex constructs. Cognitive function was measured with Raven’s matrices, which capture fluid intelligence but not other aspects like memory or attention span that scarcity might also affect. Time and risk preferences were measured through choice tasks that, although standard, are hypothetical and non-incentivised. We adopted non-incentivised elicitation for reasons of cost and feasibility, and methodological literature shows that hypothetical and incentivised elicitations typically yield similar patterns^60,61^. Relatedly, our choice of the Eckel–Grossman ordered-lottery menu over more granular alternatives such as Holt–Laury was deliberate, because the simpler menu yields less noisy responses in low-numeracy populations^62,63^. The trade-off is that it distinguishes degrees of risk aversion only up to risk neutrality and is not designed to detect risk-seeking, so our risk measure captures movement along the risk-averse-to-neutral range rather than the full spectrum of risk attitudes. Third, although we tried to control for key covariates and used random assignment for the prime, causal interpretation should be cautious. The study was not pre-registered and accordingly, we treat the region-specific tests of the primary outcomes as confirmatory and the interaction and subgroup analyses as exploratory. Future research could build on these findings by exploring interventions and moderating factors.

## Methods

### Study design and participants

This study employed a lab-in-the-field experimental design^64^ in two rural regions of China: Yunnan Province in the southwest and Shaanxi Province in the northwest. The experiment involved a between-subject design within each region, with participants randomly assigned to one of two conditions: a Hard scarcity prime or an Easy control prime. Randomisation was achieved in the field by alternating survey forms. To ensure cultural and cognitive suitability, we first conducted pilot tests in each region (16 participants in Yunnan; 14 in Shaanxi). Feedback from these pilots informed refinements to the financial scenarios and spending allocation task.

We collected data between May and September 2022, recruiting participants from local villages with the support of two local community leaders, two senior researchers, and nine trained research assistants (four from Yunnan, five from Shaanxi). Research assistants, who were well-acquainted with the local communities, recruited participants from villages and weekend markets. Inclusion criteria were: (1) 18 years of age or older; (2) residence in a rural area; and (3) ability to understand the instructions and complete the tasks.

The study protocol was approved by the Ethics Committee of Loughborough University (ID: 2022-8755-10049) and conducted in accordance with the Declaration of Helsinki (1964) and its later amendments. Participation was voluntary and anonymous, and participants were informed of their right to withdraw from the study at any time. Informed consent was obtained by participants ticking a box at the bottom of the consent information page. For individuals with limited literacy, the consent information and study instructions were explained verbally in the local dialect. All participants received a small token of appreciation (e.g., a towel) upon completion of the tasks.

### Experimental procedure and measurements

After providing informed consent, participants completed a set of experimental tasks administered by trained research assistants. Tasks were presented in a survey format and delivered orally for participants who had difficulty reading, to ensure comprehension. The survey typically took 30–45 min to complete. The complete set of instruments, the priming vignettes, the intertemporal-choice and risk tasks, the Raven’s matrices instructions, the budget-allocation task, and the demographic items, is provided in section  2 of the Supplementary Information.

To elicit scarcity feelings, we used a priming technique commonly applied in psychology and economics^65^. Participants read or heard three financial scenarios: (1) raising money in one week, (2) decline in annual income, and (3) increase in medical expenses. The first two sets were adapted from Mani et al.^21^, whereas the third set focused on healthcare financial shocks. The scenarios and questions were identical across conditions, except for the amount of money or the magnitude of financial burden. For example, the ‘hard’ scenario described an unforeseen event requiring an immediate ¥20,000 expense, whereas the ‘easy’ scenario only required ¥1000 expense. After each scenario, participants responded to both open-ended and closed-ended questions. The open-ended questions asked participants how they would handle the financial shocks (e.g., “How would you go about getting (¥20000/¥1000) on a very short notice?”) to elicit their feelings and financial concerns. The closed-ended questions were coded with five-item Likert scales to measure the levels of worries.

Immediately after the priming task, participants completed the easiest set (12 items) of the 60-item Standard Progressive Matrices, a widely used measure of fluid intelligence that does not require reading or mathematical skills, an important consideration given the low literacy levels in our study population^54^. This task assessed participants’ capacity for logical reasoning and problem-solving in novel situations^66^. Participants were encouraged to do their best, and while there was no strict time limit, they were asked to complete the task as efficiently as possible.

We then used non-incentivised methods to elicit time and risk preferences, as prior evidence suggests hypothetical monetary choices yield results comparable to incentivised ones, offering a lower-cost alternative in field experiments^67–69^. We designed two intertemporal choice tasks, one framed as receiving a cash payment and the other as receiving a health subsidy (for a physical examination), using a multiple price list (MPL) format based on the quasi-hyperbolic discounted model^35^. Each task included two sets of eight money-sooner versus money-later choices: one for today vs. one month later, and another for six months vs. seven months later (e.g., ¥200 today vs. ¥300 in one month, or ¥200 in sixth month vs. ¥300 in seventh month). The two choice scenarios were identical in terms of amount of money. Choosing the earlier, smaller payoff indicated greater impatience. Present bias (β < 1) is identified not by impatience in any single list but by the difference between the two lists—greater impatience in the immediate (today vs. next month) list than in the structurally identical delayed (six vs. seven months). Participants were considered consistent if they switched their preference at most once in each MPL (which is the pattern expected of a rational time-consistent or present-biased decision-maker), and inconsistent if they switched multiple times (which could indicate confusion or lack of stable preference). As described in the Results, we defined a participant as exhibiting present bias if they chose the smaller-sooner over the larger-later reward in the *today vs. next month* comparison but did not do so in the *six-month vs. seven-month* future comparison. The primary outcome from these tasks was the percentage of present-biased participants in Hard vs. Easy groups within each region.

We measured risk preference using the Eckel & Grossman lottery task^55^. In the monetary risk task, participants chose one among six lotteries, each lottery offering a 50/50 chance of winning either a lower or a higher monetary amount (with higher expected values for riskier lotteries). In the health-framed risk task, participants chose among six hypothetical vaccines, each offering different probabilities of short-term vs. long-term protection (analogous to the risk-return trade-offs in the monetary lotteries). Choosing a safer lottery or vaccine (one with a lower payoff range but more certain outcome) indicates greater risk aversion, whereas choosing a riskier option indicates greater risk tolerance. We recorded each participant’s choice and analysed whether the distribution of choices differed by prime condition in each region.

Next, we included a budget allocation task where participants were asked to allocate a hypothetical budget of ¥100 across three categories: daily groceries (necessities), medicine and health-related goods, and temptation goods (non-necessities such as snacks, toys or leisure items). This task was designed to mimic the experience of budgeting in a shopping scenario, aiming to make the decision process feel as realistic as possible. The specific items listed under each category were popular, familiar items in the local markets (refined based on the pilot study feedback). After participants made their allocations, we converted the amounts into percentages of the ¥100 budget for analysis.

Finally, we collected demographic and economic background information. This included age, gender, education, job, annual income, sources of income, payment frequency, physical examination, household size, household annual income and any outstanding debt. Notably, we asked participants to report the date and amount of the last payment they had received (salary, pension, remittance, etc.). Research assistants in the field helped clarify these questions and ensure accurate responses, especially for income, which some participants could only estimate.

### Statistical analysis

A sample of 301 participants were recruited from Yunnan, of which 150 were randomly assigned to the ‘hard’ scenarios and 151 to the ‘easy’ scenarios, and a sample of 245 participants were recruited from Shaanxi, of which 127 were randomly assigned to the ‘hard’ scenarios and 118 to the ‘easy’ scenarios. The sample size was determined by practical feasibility, including constraints of time and resources. A power analysis adjusted for multiple comparisons (α ≈ 0.0045) with 80% power across five primary outcomes indicated a minimum of 111 participants per group. A total of 37 participants from the Yunnan sample and 30 from the Shaanxi sample were excluded for one or more of the following reasons: incomplete survey submissions, unengaged responses, or absent information. This resulted in final samples of 264 in Yunnan (130 in the ‘hard’ group and 134 in the ‘easy’ group) and 215 in Shaanxi (110 in the ‘hard’ group and 105 in the ‘easy’ group) for the main analyses.

Data analysis was performed using STATA 17. We used Pearson chi square tests for categorical variables and t-tests for continuous variables for balancing checks comparing the demographic variations between the ‘hard’ and ‘easy’ groups. We ran separate models for Yunnan and Shaanxi and used varied regression models to examine the treatment effects on key outcome variables, including ordinary least squares (OLS) regressions for continuous variables (priming worries and cognitive function), logistic regression for binary variables (time preferences), ordered logistic regression for ordered variables (risk preferences) and seemingly unrelated regressions (SUR) for proportional variables (spending proportions). We examined the *prime × income* and *prime × days-since-last-pay* interaction terms to explore moderation by objective and fluctuation of scarcity. All regressions models used robust standard errors and controlled for demographic and income-related information. We also conducted subgroup analysis to examine the heterogeneity in the key treatment effects in Yunnan.

## Supplementary Information

Below is the link to the electronic supplementary material.

Supplementary Material 1

## Data Availability

Data will be made available in a public repository upon publication.
