# Supplementary material for: Scarcity, cognition, and health decision-making: evidence from a lab-in-the-field experiment in rural China
Source: Sci Rep. 2026 Oct 28;16:35300. doi: 10.1038/s41598-026-71861-z (PMC13719513; doi:10.1038/s41598-026-71861-z)
Supplement: Supplementary file 1 [file 41598_2026_71861_MOESM1_ESM.docx]

**Supplementary Information for**

**Scarcity, Cognition, and Health Decision-Making: Evidence from a Lab-in-the-Field Experiment in Rural China**

Haiou Zhu, Fangzhou You, E Liu, Thorsten Gruber, Hua Dong, Cees De Bont

Corresponding author: Haiou Zhu ([haiou.zhu@psych.ox.ac.uk](mailto:haiou.zhu@psych.ox.ac.uk))

**This PDF file includes:**

**Section 1. Tables S1 to S9**

**Section 2. Materials and Methods**

**Section 1. Tables S1 to S9**

**Table S1.** Participant characteristics and balance check

|  | Yunnan | | Shaanxi | | Yunnan | Shaanxi |
| --- | --- | --- | --- | --- | --- | --- |
|  | Easy  (n=130) | Hard  (n=134) | Easy  (n=110) | Hard  (n=105) | p-value | |
|  | (1) | (2) | (3) | (4) | (5) | (6) |
| *Individual Characteristics* |  |  |  |  |  |  |
| Gender |  |  |  |  | 0.22 | 0.33 |
| Male | 64 (49.2%) | 76 (56.7%) | 47 (42.7%) | 38 (36.2%) |  |  |
| Female | 66 (50.8%) | 58 (43.3%) | 63 (57.3%) | 67 (63.8%) |  |  |
| Age |  |  |  |  | 0.72 | 0.32 |
| 18-30 | 15 (11.5%) | 10 (7.5%) | 15 (13.6%) | 9 (8.6%) |  |  |
| 31-40 | 18 (13.8%) | 20 (14.9%) | 10 (9.1%) | 5 (4.8%) |  |  |
| 41-50 | 26 (20.0%) | 33 (24.6%) | 18 (16.4%) | 16 (15.2%) |  |  |
| 51-60 | 26 (20.0%) | 29 (21.6%) | 27 (24.5%) | 37 (35.2%) |  |  |
| 61 and above | 45 (34.6%) | 42 (31.3%) | 40 (36.4%) | 38 (36.2%) |  |  |
| Education |  |  |  |  | 0.30 | 0.002*** |
| Illiterate | 29 (22.3%) | 20 (14.9%) | 7 (6.4%) | 22 (21.0%) |  |  |
| Primary school | 49 (37.7%) | 52 (38.8%) | 29 (26.4%) | 34 (32.4%) |  |  |
| Middle school | 24 (18.5%) | 28 (20.9%) | 56 (50.9%) | 43 (41.0%) |  |  |
| High school | 11 (8.5%) | 20 (14.9%) | 11 (10.0%) | 5 (4.8%) |  |  |
| Undergraduate and above | 17 (13.1%) | 14 (10.4%) | 7 (6.4%) | 1 (1.0%) |  |  |
| Source of Income |  |  |  |  | 0.027** | 0.24 |
| Migrant Works | 35 (26.9%) | 49 (36.6%) | 40 (36.4%) | 47 (44.8%) |  |  |
| Farming | 24 (18.5%) | 21 (15.7%) | 31 (28.2%) | 21 (20.0%) |  |  |
| No Income | 30 (23.1%) | 14 (10.4%) | 24 (21.8%) | 17 (16.2%) |  |  |
| Other sources | 41 (31.5%) | 50 (37.3%) | 15 (13.6%) | 20 (19.0%) |  |  |
| Frequency of Payments |  |  |  |  | 0.32 | 0.33 |
| Monthly | 64 (49.2%) | 78 (58.2%) | 15 (13.6%) | 19 (18.1%) |  |  |
| Every Three/Six Months | 4 (3.1%) | 1 (0.7%) | 16 (14.5%) | 5 (10.5%) |  |  |
| Annually | 4 (3.1%) | 4 (3.0%) | 8 (7.3%) | 12 (11.4%) |  |  |
| Unfixed | 58 (44.6%) | 51 (38.1%) | 71 (64.5%) | 63 (60.0%) |  |  |
| Annual income | 25528.40 (28328.93) | 30416.56 (27421.11) | 9916.20 (14670.67) | 7477.33 (10176.88) | 0.16 | 0.17 |
| Days since last payment | 31.69 (30.30) | 31.05 (48.69) | 96.28 (75.24) | 93.03 (73.41) | 0.90 | 0.79 |
| Amount of last payment | 3162.50 (3113.28) | 2826.39 (2851.43) | 2592.36 (5722.44) | 2328.86 (2941.57) | 0.36 | 0.67 |
| *Household Characteristics* |  |  |  |  |  |  |
| Household size | 4.84 (1.67) | 4.66 (1.80) | 4.64 (1.52) | 4.70 (2.00) | 0.40 | 0.78 |
| Annual household income | 57370.00 (38076.88) | 61505.12 (41114.03) | 25043.06 (26902.27) | 23050.00 (22942.83) | 0.40 | 0.56 |
| Annual household income per adult | 26502.79 (18122.71) | 28813.07 (19241.53) | 11703.18 (12568.07) | 10881.14 (10515.08) | 0.32 | 0.61 |
| Household debt (>50% of annual household income) |  |  |  |  | 0.73 | 0.12 |
| Yes | 60 (46.2%) | 59 (44.0%) | 49 (44.5%) | 36 (34.3%) |  |  |
| No | 70 (53.8%) | 75 (56.0%) | 61 (55.5%) | 69 (65.7%) |  |  |
| Physical examination |  |  |  |  | 0.66 | 0.04** |
| Yes | 82 (63.1%) | 88 (65.7%) | 38 (34.5%) | 23 (21.9%) |  |  |
| No | 48 (36.9%) | 46 (34.3%) | 72 (65.5%) | 82 (78.1%) |  |  |

**Table 1.** Participant characteristics and balance check. Note: Pearson's chi-squared test for binary variables, Fisher's exact test for categorizable variables and two sample t test for continuous variables. All variables include full samples (Yunnan: n=264; Shaanxi: n=215) except for the following variables: days since last payment and amount of last payment restricted to subjects who report their days since payment fewer than 365 days (Yunnan: n=262; Shaanxi: n=154). Annual household income per adult is computed by dividing total annual household income by the square root of household size.

**Table S2**. Manipulation check

| Scenarios and questions | Mean for  Hard Scenario | | {Easy Scenario} | |  | |  |
| --- | --- | --- | --- | --- | --- | --- | --- |
|  |  |  | Coefficient | | P-value | | |
|  | Yunnan | Shaanxi | Yunnan | Shaanxi | Yunnan | Shaanxi | |
|  | (1) | (2) | (3) | (4) | (5) | | (6) |
| **Scenario 1: Raise money in one week**.  Imagine that an unforeseen event requires of you an immediate (¥20000/¥1000) expense. You need to raise the money in less than a week. |  |  |  |  |  | |  |
| Are there ways in which you may be able to raise the money in one week? (Yes/No) | 1.43 | 1.72 | -.27 | -.39 | <.001*** | | <.001*** |
| To what extent do you agree with the following statements:  (a) "Coming up with (¥20000/¥1000) on a very short notice would cause me long-lasting ﬁnancial hardship.” | 2.99 | 3.76 | -.52 | -.60 | .007** | | .003** |
| (b) "Coming up with (¥20000/¥1000) on a very short notice would require me to make sacriﬁces that have long-term consequences.” | 3.24 | 3.93 | -.72 | -.39 | <.001*** | | .041** |
| **Scenario 2: Income decrease shock.**  Imagine that the economy is going through diﬃcult times. The income in your family decreases by (50%/5%). To what extent do you agree with the following statements: |  |  |  |  |  | |  |
| (a) "Given my situation, I would be able to maintain roughly the same lifestyle under those new circumstances.” | 2.11 | 2.70 | 1.51 | .56 | <.001*** | | .003** |
| (b) "The (50%/5%) decrease in our income would strongly impact our daily life." | 3.95 | 3.95 | -1.64 | -.83 | <.001*** | | <.001*** |
| **Scenario 3 Healthcare increase shock**.  Imaging that due to serve illness, there is an increase in the monthly cost of healthcare by (¥2000/¥100) for your family, which amounts to a total cost increase of (¥24000/¥1200) a year. This increase is not reimbursable by any government funding scheme. |  |  |  |  |  | |  |
| Would it be diﬃcult to aﬀord healthcare? (Yes/No) | 1.32 | 1.10 | .44 | .31 | <.001*** | | <.001*** |
| To what extent do you agree with the following statements:  (a) "Paying additional (¥2000/¥100) a month for healthcare would require diﬃcult budget cuts and sacriﬁces every month.” | 3.87 | 3.89 | -1.42 | -.92 | <.001*** | | <.001*** |
| (b) "Paying additional (¥2000/¥100) a month for healthcare would be too costly and it would probably result in forgoing going to the hospital.” | 3.46 | 3.86 | -1.09 | -.88 | <.001*** | | <.001*** |
| Joint test |  |  |  |  | <.001*** | | <.001*** |

Notes: The table investigates hard-easy differences in the primed financial worries measured with 5-item Likert scale, except for two questions with dichotomy answers (yes/no). Columns (1)-(2) show means of perceived worries for the Hard group and Columns (3)-(4) show the coefficient between the Hard and Easy groups. Columns (5)-(6) report the *p*-value of Pearson chi square test for dichotomous questions or t-tests for Likert scale measurements; *** p<0.01, ** p<0.05, * p<0.1.

**Table S3**. Effects of priming, annual income and distance to payday on cognitive function

|  | Raven's Progressive Matrices Accuracy | | | | | |
| --- | --- | --- | --- | --- | --- | --- |
|  | Yunnan | | | Shaanxi | | |
| Hard | -8.40^***^ | -8.63^***^ | -7.76^**^ | 1.20 | 3.29 | 1.56 |
|  | (2.81) | (2.88) | (3.58) | (3.63) | (4.18) | (4.92) |
| Annual income |  | -1.83 |  |  | 2.63 |  |
|  |  | (2.68) |  |  | (3.99) |  |
| Hard # Annual income |  | 1.50 |  |  | 3.03 |  |
|  |  | (2.43) |  |  | (5.55) |  |
| Days since last payment |  |  | -3.04 |  |  | -1.98 |
|  |  |  | (5.68) |  |  | (2.93) |
| Hard # Days since last payment |  |  | 0.90 |  |  | 1.30 |
|  |  |  | (5.66) |  |  | (3.72) |
| Constant | 76.92^***^ | 75.96^***^ | 74.56^***^ | 62.10^***^ | 60.32^***^ | 61.17^***^ |
|  | (7.65) | (7.56) | (8.87) | (9.48) | (10.33) | (12.13) |
| Observations | 264 | 263 | 262 | 215 | 209 | 154 |
| Adjusted *R*^2^ | 0.471 | 0.474 | 0.464 | 0.218 | 0.202 | 0.249 |
| Controls | Yes | Yes | Yes | Yes | Yes | Yes |

Standard errors in parentheses

Robust standard errors reported. *** p<0.01, ** p<0.05, * p<0.1

**Table S4.1** Descriptive results for all outcome variables (Yunnan)

| Factor | Easy | Hard | p-value | Test |
| --- | --- | --- | --- | --- |
| N | 130 | 134 |  |  |
| Worry about raising money within a week (Yes/No) | 21 (16.2%) | 58 (43.3%) | <0.001 | Pearson's chi-squared |
| Mean value of Worry1, mean (SD) | 2.50 (1.36) | 3.12 (1.57) | <0.001 | Two sample t |
| Mean value of Worry2, mean (SD) | 2.34 (1.00) | 3.92 (1.19) | <0.001 | Two sample t |
| Worry about paying for medical expenses (Yes/No) | 31 (23.8%) | 91 (67.9%) | <0.001 | Pearson's chi-squared |
| Mean value of Worry3, mean (SD) | 2.41 (1.25) | 3.66 (1.28) | <0.001 | Two sample t |
| Total Worry Score, mean (SD) | 2.42 (1.01) | 3.57 (1.12) | <0.001 | Two sample t |
| Raven's Progressive Matrices Score, mean (SD) | 7.38 (3.60) | 6.55 (3.61) | 0.062 | Two sample t |
| Raven's Progressive Matrices Accuracy, mean (SD) | 61.54 (29.97) | 54.60 (30.12) | 0.062 | Two sample t test |
| Monetary Risk Assessment |  |  | 0.10 | Fisher's exact |
| 1 | 58 (44.6%) | 66 (49.3%) |  |  |
| 2 | 18 (13.8%) | 13 (9.7%) |  |  |
| 3 | 13 (10.0%) | 12 (9.0%) |  |  |
| 4 | 18 (13.8%) | 7 (5.2%) |  |  |
| 5 | 6 (4.6%) | 8 (6.0%) |  |  |
| 6 | 17 (13.1%) | 28 (20.9%) |  |  |
| Health Risk Assessment |  |  | 0.005 | Fisher's exact |
| Vaccine1 | 84 (64.6%) | 110 (82.1%) |  |  |
| Vaccine2 | 18 (13.8%) | 4 (3.0%) |  |  |
| Vaccine3 | 11 (8.5%) | 6 (4.5%) |  |  |
| Vaccine4 | 6 (4.6%) | 6 (4.5%) |  |  |
| Vaccine6 | 11 (8.5%) | 8 (6.0%) |  |  |
| Present Bias Monetary |  |  | 0.12 | Fisher's exact |
| Present bias | 69 (53.1%) | 64 (47.8%) |  |  |
| Not bias | 56 (43.1%) | 69 (51.5%) |  |  |
| Inconsistent | 5 (3.8%) | 1 (0.7%) |  |  |
| BiasM_b | 69 (55.2%) | 64 (48.1%) | 0.26 | Pearson's chi-squared |
| Present Bias Health |  |  | 0.69 | Fisher's exact |
| Present bias | 49 (37.7%) | 51 (38.1%) |  |  |
| No bias | 73 (56.2%) | 78 (58.2%) |  |  |
| Inconsistent | 8 (6.2%) | 5 (3.7%) |  |  |
| BiasH_b | 49 (40.2%) | 51 (39.5%) | 0.92 | Pearson's chi-squared |
| Proportion of Grocery Expenditure, mean (SD) | 0.60 (0.22) | 0.58 (0.21) | 0.47 | Two sample t test |
| Proportion of Health Expenditure, mean (SD) | 0.29 (0.21) | 0.33 (0.19) | 0.064 | Two sample t test |
| Proportion of Temptation Expenditure, mean (SD) | 0.12 (0.18) | 0.09 (0.15) | 0.18 | Two sample t test |
| Total Spending, mean (SD) | 90.72 (11.98) | 98.39 (7.38) | <0.001 | Two sample t test |

**Table S4.2** Descriptive results for all outcome variables (Shaanxi)

| Factor | Easy | Hard | p-value | Test |
| --- | --- | --- | --- | --- |
| N | 110 | 105 |  |  |
| Worry about raising money within a week (Yes/No) | 37 (33.6%) | 76 (72.4%) | <0.001 | Pearson's chi-squared |
| Mean value of Worry1, mean (SD) | 3.35 (1.39) | 3.85 (1.30) | 0.008 | Two sample t test |
| Mean value of Worry2, mean (SD) | 2.93 (0.90) | 3.63 (0.94) | <0.001 | Two sample t test |
| Worry about paying for medical expenses (Yes/No) | 66 (60.0%) | 95 (90.5%) | <0.001 | Pearson's chi-squared |
| Mean value of Worry3, mean (SD) | 2.97 (1.35) | 3.87 (1.33) | <0.001 | Two sample t test |
| Total Worry Score, mean (SD) | 3.08 (0.93) | 3.78 (0.95) | <0.001 | Two sample t test |
| Raven's Progressive Matrices Score, mean (SD) | 7.23 (3.36) | 7.01 (2.74) | 0.60 | Two sample t test |
| Raven's Progressive Matrices Accuracy, mean (SD) | 60.23 (28.01) | 58.41 (22.84) | 0.60 | Two sample t test |
| Monetary Risk Assessment |  |  | 0.42 | Fisher's exact |
| 1 | 53 (48.2%) | 52 (49.5%) |  |  |
| 2 | 11 (10.0%) | 7 (6.7%) |  |  |
| 3 | 18 (16.4%) | 16 (15.2%) |  |  |
| 4 | 8 (7.3%) | 7 (6.7%) |  |  |
| 5 | 9 (8.2%) | 4 (3.8%) |  |  |
| 6 | 11 (10.0%) | 19 (18.1%) |  |  |
| Health Risk Assessment |  |  | 0.46 | Fisher's exact |
| Vaccine1 | 58 (52.7%) | 52 (49.5%) |  |  |
| Vaccine2 | 8 (7.3%) | 17 (16.2%) |  |  |
| Vaccine3 | 13 (11.8%) | 12 (11.4%) |  |  |
| Vaccine4 | 5 (4.5%) | 5 (4.8%) |  |  |
| Vaccine5 | 5 (4.5%) | 3 (2.9%) |  |  |
| Vaccine6 | 21 (19.1%) | 16 (15.2%) |  |  |
| Present Bias Monetary |  |  | 0.66 | Fisher's exact |
| Present bias | 31 (28.2%) | 32 (30.5%) |  |  |
| No bias | 59 (53.6%) | 50 (47.6%) |  |  |
| Inconsistent | 20 (18.2%) | 23 (21.9%) |  |  |
| BiasM_b | 31 (34.4%) | 32 (39%) | 0.53 | Pearson's chi-squared |
| Present Bias Health |  |  | 0.85 | Fisher's exact |
| Present bias | 32 (29.1%) | 27 (25.7%) |  |  |
| No bias | 64 (58.2%) | 65 (61.9%) |  |  |
| Inconsistent | 14 (12.7%) | 13 (12.4%) |  |  |
| BiasH_b | 32 (33.3%) | 27 (29.3%) | 0.56 | Pearson's chi-squared |
| Proportion of Grocery Expenditure, mean (SD) | 0.68 (0.22) | 0.69 (0.22) | 0.74 | Two sample t test |
| Proportion of Health Expenditure, mean (SD) | 0.21 (0.16) | 0.17 (0.14) | 0.046 | Two sample t test |
| Proportion of Temptation Expenditure, mean (SD) | 0.11 (0.13) | 0.14 (0.16) | 0.12 | Two sample t test |
| Total Spending, mean (SD) | 98.61 (4.21) | 96.36 (2.68) | <0.001 | Two sample t test |

**Table S5.1.** Effects of priming, annual income and distance to payday on time preferences (present bias)

|  | Time preferences (present bias) | | | | | | | | | | | |
| --- | --- | --- | --- | --- | --- | --- | --- | --- | --- | --- | --- | --- |
|  | Yunnan | | | | | | Shaanxi | | | | | |
|  | Monetary choices | | | Health choices | | | Monetary choices | | | Health choices | | |
| Hard | -0.52^*^ | -0.58^*^ | -0.50 | -0.02 | -0.08 | -0.24 | 0.00 | -0.15 | -0.11 | -0.28 | -0.21 | 0.00 |
|  | (0.28) | (0.30) | (0.34) | (0.28) | (0.30) | (0.36) | (0.39) | (0.49) | (0.55) | (0.37) | (0.53) | (0.53) |
| Annual income |  | -0.06 |  |  | -0.26 |  |  | 0.04 |  |  | 0.66 |  |
|  |  | (0.21) |  |  | (0.22) |  |  | (0.55) |  |  | (0.53) |  |
| Hard # Annual income |  | 0.09 |  |  | 0.27 |  |  | -0.52 |  |  | 0.12 |  |
|  |  | (0.23) |  |  | (0.24) |  |  | (0.70) |  |  | (0.78) |  |
| Days since last payment |  |  | 0.00 |  |  | 0.15 |  |  | -0.03 |  |  | 0.08 |
|  |  |  | (0.45) |  |  | (0.48) |  |  | (0.36) |  |  | (0.26) |
| Hard # Days since last payment |  |  | 0.21 |  |  | -0.49 |  |  | 0.04 |  |  | 0.01 |
|  |  |  | (0.49) |  |  | (0.53) |  |  | (0.40) |  |  | (0.35) |
| Constant | 2.20^**^ | 2.26^***^ | 2.28^**^ | 0.35 | 0.27 | 0.37 | -1.83 | -1.10 | -1.62 | 0.32 | 0.07 | 1.60 |
|  | (0.86) | (0.87) | (0.92) | (0.89) | (0.89) | (0.93) | (1.39) | (1.37) | (2.02) | (1.08) | (1.16) | (1.51) |
| Observations | 258 | 257 | 256 | 251 | 250 | 249 | 172 | 168 | 126 | 188 | 183 | 130 |
| Pseudo R-squared | 0.063 | 0.067 | 0.070 | 0.069 | 0.073 | 0.076 | 0.139 | 0.162 | 0.143 | 0.099 | 0.104 | 0.133 |

Standard errors in parentheses. Robust standard errors reported. *** p<0.01, ** p<0.05, * p<0.1

**Table S5.2.** OLS regressions of the continuous discounting-ratio measure (β) on the scarcity prime, including inconsistent decision-makers (full sample)

|  | **Monetary (β, money)** | | **Health (β, health)** | |
| --- | --- | --- | --- | --- |
|  | **Yunnan** | **Shaanxi** | **Yunnan** | **Shaanxi** |
| Hard prime | 0.009 | 0.013 | −0.014 | −0.014 |
|  | (0.012) | (0.025) | (0.013) | (0.016) |
| 95% CI | [−0.014, 0.032] | [−0.037, 0.063] | [−0.039, 0.011] | [−0.045, 0.017] |
| P-value | 0.434 | 0.612 | 0.261 | 0.378 |
| Controls | Yes | Yes | Yes | Yes |
| Observations | 264 | 215 | 264 | 215 |
| R² | 0.119 | 0.068 | 0.109 | 0.148 |

**Notes:** The dependent variable is the continuous discounting-ratio measure underlying the present-bias classification (β), computed from the switch points in the two multiple price lists for the monetary task (TimeM) and the health task (TimeH); β < 1 indicates present bias, β = 1 no bias, and β > 1 a reversed (inconsistent) pattern. “Hard prime” is an indicator for assignment to the Hard scarcity condition (reference: Easy). All models include the standard control set used throughout the paper: gender, age group, education, source of income, payment frequency, recent health exam, standardised amount of last payment, standardised household annual income, and standardised household size. Estimation is by ordinary least squares with heteroskedasticity-robust (HC1) standard errors, shown in parentheses.

**Table S6.** Effects of priming, annual income and distance to payday on risk preferences

|  | Risk preferences | | | | | | | | | | | |
| --- | --- | --- | --- | --- | --- | --- | --- | --- | --- | --- | --- | --- |
|  | Yunnan | | | | | | Shaanxi | | | | | |
|  | Monetary choices | | | Health choices | | | Monetary choices | | | Health choices | | |
| Hard | -0.16 | -0.21 | -0.29 | -0.84^***^ | -1.10^***^ | -1.22^***^ | 0.30 | 0.40 | 0.58 | 0.11 | 0.02 | 0.46 |
|  | (0.26) | (0.30) | (0.40) | (0.31) | (0.37) | (0.41) | (0.32) | (0.46) | (0.47) | (0.32) | (0.49) | (0.49) |
| Annual income |  | 0.20 |  |  | -0.15 |  |  | -0.19 |  |  | -0.65 |  |
|  |  | (0.18) |  |  | (0.24) |  |  | (0.44) |  |  | (0.51) |  |
| Hard # Annual income |  | 0.06 |  |  | 0.41 |  |  | 0.38 |  |  | 0.12 |  |
|  |  | (0.23) |  |  | (0.31) |  |  | (0.70) |  |  | (0.70) |  |
| Days since last payment |  |  | -0.37 |  |  | 0.95^*^ |  |  | 0.10 |  |  | 0.41 |
|  |  |  | (0.35) |  |  | (0.52) |  |  | (0.18) |  |  | (0.26) |
| Hard # Days since last payment |  |  | -0.33 |  |  | -0.84 |  |  | -0.38 |  |  | -0.77^**^ |
|  |  |  | (0.71) |  |  | (0.55) |  |  | (0.33) |  |  | (0.32) |
| / |  |  |  |  |  |  |  |  |  |  |  |  |
| cut1 | -1.82^**^ | -1.79^**^ | -1.52^*^ | -0.14 | -0.08 | -0.63 | -1.30^*^ | -1.25 | -1.67 | 1.47 | 0.92 | 1.89^*^ |
|  | (0.84) | (0.87) | (0.83) | (0.90) | (0.94) | (0.93) | (0.75) | (0.84) | (1.02) | (0.91) | (0.94) | (1.09) |
| cut2 | -1.21 | -1.18 | -0.91 | 0.42 | 0.49 | -0.08 | -0.91 | -0.84 | -1.27 | 2.02^**^ | 1.48 | 2.30^**^ |
|  | (0.84) | (0.87) | (0.83) | (0.89) | (0.93) | (0.92) | (0.75) | (0.84) | (1.02) | (0.91) | (0.94) | (1.07) |
| cut3 | -0.70 | -0.67 | -0.39 | 0.98 | 1.07 | 0.50 | -0.11 | -0.03 | -0.63 | 2.62^***^ | 2.11^**^ | 2.88^***^ |
|  | (0.85) | (0.87) | (0.84) | (0.90) | (0.94) | (0.93) | (0.75) | (0.84) | (1.01) | (0.90) | (0.93) | (1.07) |
| cut4 | -0.12 | -0.11 | 0.17 | 1.56^*^ | 1.61^*^ | 1.08 | 0.30 | 0.40 | -0.24 | 2.90^***^ | 2.40^***^ | 3.24^***^ |
|  | (0.84) | (0.87) | (0.83) | (0.94) | (0.97) | (0.97) | (0.75) | (0.84) | (1.01) | (0.90) | (0.93) | (1.07) |
| cut5 | 0.26 | 0.28 | 0.56 |  |  |  | 0.75 | 0.84 | 0.23 | 3.16^***^ | 2.67^***^ | 3.52^***^ |
|  | (0.85) | (0.88) | (0.84) |  |  |  | (0.76) | (0.85) | (1.03) | (0.90) | (0.92) | (1.07) |
| Observations | 264 | 263 | 262 | 264 | 263 | 262 | 215 | 209 | 154 | 215 | 209 | 154 |
| Pseudo R-squared | 0.095 | 0.097 | 0.099 | 0.076 | 0.080 | 0.083 | 0.050 | 0.051 | 0.060 | 0.056 | 0.057 | 0.116 |

Standard errors in parentheses

Robust standard errors reported. *** p<0.01, ** p<0.05, * p<0.1

**Table S7.1** SUR regression results
All SUR models reported joint significance of system equations (χ² test) and met standard robustness requirements. Observations ranged from n = 136 to n = 192 per region. All P values are two-tailed and exact. Variables were standardised prior to analysis, and model assumptions were assessed via residual diagnostics. Data were approximately normally distributed, justifying use of linear estimators.

Effects of priming, annual income and distance to payday on purchase decisions (Yunnan) using OLS regression

|  | (1) | (2) | (3) | (4) | (5) | (6) | (7) | (8) | (9) |
| --- | --- | --- | --- | --- | --- | --- | --- | --- | --- |
| Hard | -0.03 | -0.05 | -0.14 | 0.18 | 0.21 | 0.22 | -0.18 | -0.18 | -0.08 |
|  | (0.13) | (0.12) | (0.14) | (0.14) | (0.13) | (0.15) | (0.13) | (0.11) | (0.12) |
| Annual income |  | -0.10 |  |  | 0.10 |  |  | 0.03 |  |
|  |  | (0.09) |  |  | (0.11) |  |  | (0.09) |  |
| Hard # Annual income |  | 0.09 |  |  | -0.05 |  |  | -0.06 |  |
|  |  | (0.09) |  |  | (0.12) |  |  | (0.10) |  |
| Days since last payment |  |  | 0.05 |  |  | 0.19 |  |  | -0.31^**^ |
|  |  |  | (0.15) |  |  | (0.16) |  |  | (0.15) |
| Hard # Days since last payment |  |  | -0.27 |  |  | 0.11 |  |  | 0.24 |
|  |  |  | (0.20) |  |  | (0.23) |  |  | (0.15) |
| Constant | -0.41 | -0.45 | -0.42 | 0.01 | 0.01 | 0.16 | 0.57 | 0.63^*^ | 0.39 |
|  | (0.34) | (0.34) | (0.35) | (0.30) | (0.30) | (0.31) | (0.37) | (0.37) | (0.38) |
| Observations | 264 | 263 | 262 | 264 | 263 | 262 | 264 | 263 | 262 |
| R-squared | 0.079 | 0.088 | 0.094 | 0.138 | 0.139 | 0.160 | 0.175 | 0.181 | 0.183 |

**Table S7.2** Effects of priming, annual income and distance to payday on purchase decisions (Shaanxi) using OLS regressions

|  | (1) | (2) | (3) | (4) | (5) | (6) | (7) | (8) | (9) |
| --- | --- | --- | --- | --- | --- | --- | --- | --- | --- |
| Hard | -0.14 | -0.42^*^ | -0.21 | -0.14 | 0.10 | -0.01 | 0.37^**^ | 0.48^**^ | 0.32^*^ |
|  | (0.14) | (0.23) | (0.19) | (0.11) | (0.18) | (0.15) | (0.14) | (0.20) | (0.19) |
| Annual income |  | 0.33 |  |  | -0.22 |  |  | -0.19 |  |
|  |  | (0.24) |  |  | (0.21) |  |  | (0.18) |  |
| Hard # Annual income |  | -0.57^*^ |  |  | 0.44 |  |  | 0.26 |  |
|  |  | (0.32) |  |  | (0.27) |  |  | (0.27) |  |
| Days since last payment |  |  | -0.03 |  |  | -0.02 |  |  | 0.06 |
|  |  |  | (0.10) |  |  | (0.08) |  |  | (0.09) |
| Hard # Days since last payment |  |  | 0.11 |  |  | -0.05 |  |  | -0.09 |
|  |  |  | (0.13) |  |  | (0.10) |  |  | (0.13) |
| Constant | 0.50 | 0.86^*^ | 0.77 | -0.17 | -0.48 | -0.14 | -0.51 | -0.63 | -0.92 |
|  | (0.44) | (0.50) | (0.61) | (0.34) | (0.37) | (0.43) | (0.46) | (0.53) | (0.62) |
| Observations | 191 | 185 | 142 | 191 | 185 | 142 | 191 | 185 | 142 |
| R-squared | 0.171 | 0.207 | 0.177 | 0.160 | 0.186 | 0.199 | 0.185 | 0.200 | 0.197 |

**Table S8.1** Subgroup Analysis of Cognitive Function (Yunnan)

|  | No  income | Unfixed  income | Lower Income | Days | Last pay | Debt | Gender | Age | Education | Household number | Health exam |
| --- | --- | --- | --- | --- | --- | --- | --- | --- | --- | --- | --- |
|  | b/se/p | b/se/p | b/se/p | b/se/p | b/se/p | b/se/p | b/se/p | b/se/p | b/se/p | b/se/p | b/se/p |
| Hard | -25.58 | -10.25 | -9.64 | -12.35 | -16.77 | -5.22 | -10.70 | -9.43 | -2.03 | -6.62 | -10.72 |
|  | 9.44 | 4.69 | 3.93 | 4.28 | 3.99 | 4.68 | 4.20 | 3.85 | 4.96 | 4.41 | 5.40 |
|  | 0.01 | 0.03 | 0.02 | 0.00 | 0.00 | 0.27 | 0.01 | 0.02 | 0.68 | 0.14 | 0.05 |
| _cons | 78.96 | 69.54 | 64.45 | 68.74 | 57.91 | 73.87 | 82.88 | 61.10 | 96.88 | 82.42 | 62.80 |
|  | 14.89 | 13.97 | 16.27 | 11.65 | 16.10 | 12.62 | 12.73 | 10.59 | 8.90 | 13.51 | 17.74 |
|  | 0.00 | 0.00 | 0.00 | 0.00 | 0.00 | 0.00 | 0.00 | 0.00 | 0.00 | 0.00 | 0.00 |
| Observations | 44 | 122 | 159 | 129 | 136 | 119 | 124 | 142 | 62 | 144 | 94 |
| R-squared | 0.57 | 0.47 | 0.53 | 0.50 | 0.49 | 0.47 | 0.60 | 0.38 | 0.47 | 0.42 | 0.57 |
| Controls | Yes | Yes | Yes | Yes | Yes | Yes | Yes | Yes | Yes | Yes | Yes |

Robust standard errors reported. *** p<0.01, ** p<0.05, * p<0.1

**Table S8.2** Subgroup Analysis of Grocery Proportion (Yunnan)

|  | No  income | Unfixed  income | Lower Income | Days | Last pay | Debt | Gender | Age | Education | Household number | Health exam |
| --- | --- | --- | --- | --- | --- | --- | --- | --- | --- | --- | --- |
|  | b/se/p | b/se/p | b/se/p | b/se/p | b/se/p | b/se/p | b/se/p | b/se/p | b/se/p | b/se/p | b/se/p |
| Hard | -0.12 | -0.01 | -0.02 | -0.06 | -0.07 | -0.01 | -0.06 | -0.05 | 0.02 | 0.01 | 0.02 |
|  | 0.08 | 0.04 | 0.03 | 0.04 | 0.03 | 0.04 | 0.04 | 0.03 | 0.07 | 0.04 | 0.05 |
|  | 0.13 | 0.86 | 0.57 | 0.13 | 0.04 | 0.73 | 0.09 | 0.12 | 0.77 | 0.81 | 0.67 |
| _cons | 0.73 | 0.40 | 0.47 | 0.41 | 0.67 | 0.75 | 0.46 | 0.71 | 0.42 | 0.37 | 0.40 |
|  | 0.19 | 0.16 | 0.14 | 0.12 | 0.12 | 0.10 | 0.11 | 0.08 | 0.13 | 0.15 | 0.14 |
|  | 0.00 | 0.01 | 0.00 | 0.00 | 0.00 | 0.00 | 0.00 | 0.00 | 0.00 | 0.01 | 0.01 |
| Observations | 44 | 122 | 159 | 129 | 136 | 119 | 124 | 142 | 62 | 144 | 94 |
| R-squared | 0.42 | 0.22 | 0.16 | 0.23 | 0.21 | 0.24 | 0.22 | 0.15 | 0.18 | 0.12 | 0.14 |
| Controls | Yes | Yes | Yes | Yes | Yes | Yes | Yes | Yes | Yes | Yes | Yes |

Robust standard errors reported. *** p<0.01, ** p<0.05, * p<0.1

**Table S8.3** Subgroup Analysis of Health Proportion (Yunnan)

|  | No  income | Unfixed  income | Lower Income | Days | Last pay | Debt | Gender | Age | Education | Household number | Health exam |
| --- | --- | --- | --- | --- | --- | --- | --- | --- | --- | --- | --- |
|  | b/se/p | b/se/p | b/se/p | b/se/p | b/se/p | b/se/p | b/se/p | b/se/p | b/se/p | b/se/p | b/se/p |
| Hard | 0.12 | -0.00 | 0.05 | 0.09 | 0.07 | 0.05 | 0.06 | 0.05 | -0.00 | 0.05 | 0.04 |
|  | 0.08 | 0.04 | 0.03 | 0.04 | 0.03 | 0.04 | 0.03 | 0.03 | 0.07 | 0.04 | 0.04 |
|  | 0.16 | 0.95 | 0.09 | 0.02 | 0.03 | 0.20 | 0.08 | 0.11 | 0.98 | 0.14 | 0.30 |
| _cons | 0.05 | 0.42 | 0.27 | 0.29 | 0.04 | 0.14 | 0.32 | 0.22 | 0.45 | 0.30 | 0.35 |
|  | 0.18 | 0.13 | 0.11 | 0.10 | 0.12 | 0.09 | 0.08 | 0.10 | 0.10 | 0.11 | 0.09 |
|  | 0.78 | 0.00 | 0.02 | 0.01 | 0.75 | 0.14 | 0.00 | 0.03 | 0.00 | 0.01 | 0.00 |
| Observations | 44 | 122 | 159 | 129 | 136 | 119 | 124 | 142 | 62 | 144 | 94 |
| R-squared | 0.45 | 0.24 | 0.28 | 0.25 | 0.23 | 0.24 | 0.23 | 0.17 | 0.20 | 0.21 | 0.35 |
| Controls | Yes | Yes | Yes | Yes | Yes | Yes | Yes | Yes | Yes | Yes | Yes |

Robust standard errors reported. *** p<0.01, ** p<0.05, * p<0.1

**Table S8.4** Subgroup Analysis of Temptation Proportion (Yunnan)

|  | No  income | Unfixed  income | Lower Income | Days | Last pay | Debt | Gender | Age | Education | Household number | Health exam |
| --- | --- | --- | --- | --- | --- | --- | --- | --- | --- | --- | --- |
|  | b/se/p | b/se/p | b/se/p | b/se/p | b/se/p | b/se/p | b/se/p | b/se/p | b/se/p | b/se/p | b/se/p |
| Group | 0.01 | 0.01 | -0.03 | -0.03 | -0.01 | -0.03 | 0.00 | -0.00 | -0.02 | -0.06 | -0.06 |
|  | 0.04 | 0.03 | 0.02 | 0.03 | 0.02 | 0.03 | 0.02 | 0.02 | 0.04 | 0.03 | 0.04 |
|  | 0.88 | 0.74 | 0.09 | 0.34 | 0.74 | 0.29 | 0.96 | 0.98 | 0.65 | 0.04 | 0.19 |
| _cons | 0.22 | 0.17 | 0.26 | 0.30 | 0.29 | 0.12 | 0.22 | 0.07 | 0.13 | 0.32 | 0.25 |
|  | 0.11 | 0.09 | 0.13 | 0.09 | 0.09 | 0.09 | 0.08 | 0.06 | 0.08 | 0.11 | 0.12 |
|  | 0.05 | 0.06 | 0.05 | 0.00 | 0.00 | 0.18 | 0.00 | 0.25 | 0.13 | 0.00 | 0.05 |
| Observations | 44 | 122 | 159 | 129 | 136 | 119 | 124 | 142 | 62 | 144 | 94 |
| R-squared | 0.61 | 0.32 | 0.30 | 0.30 | 0.41 | 0.16 | 0.33 | 0.25 | 0.23 | 0.23 | 0.31 |
| Controls | Yes | Yes | Yes | Yes | Yes | Yes | Yes | Yes | Yes | Yes | Yes |

Robust standard errors reported. *** p<0.01, ** p<0.05, * p<0.1

**Table S9.1** Subgroup Analysis of Cognition (Shaanxi)

|  | No  income | Unfixed  income | Lower Income | Days | Last pay | Debt | Gender | Age | Education | Household number | Health exam |
| --- | --- | --- | --- | --- | --- | --- | --- | --- | --- | --- | --- |
|  | b/se/p | b/se/p | b/se/p | b/se/p | b/se/p | b/se/p | b/se/p | b/se/p | b/se/p | b/se/p | b/se/p |
| Group | 3.07 | -1.00 | 2.96 | 0.64 | 2.13 | -4.31 | 2.77 | 5.44 | 0.01 | -1.33 | 2.78 |
|  | 12.54 | 3.99 | 3.95 | 3.89 | 4.62 | 4.84 | 5.36 | 4.98 | 6.08 | 4.94 | 4.51 |
|  | 0.81 | 0.80 | 0.46 | 0.87 | 0.65 | 0.38 | 0.61 | 0.28 | 1.00 | 0.79 | 0.54 |
| _cons | 90.93 | 61.44 | 61.79 | 60.97 | 53.73 | 58.85 | 56.70 | 39.40 | 37.16 | 68.26 | 61.13 |
|  | 35.80 | 12.24 | 12.87 | 10.88 | 16.54 | 19.31 | 12.98 | 12.17 | 25.23 | 12.95 | 11.41 |
|  | 0.02 | 0.00 | 0.00 | 0.00 | 0.00 | 0.00 | 0.00 | 0.00 | 0.24 | 0.00 | 0.00 |
| Observations | 41 | 181 | 186 | 204 | 145 | 85 | 130 | 142 | 24 | 129 | 154 |
| R-squared | 0.53 | 0.31 | 0.25 | 0.29 | 0.26 | 0.44 | 0.27 | 0.18 | 0.94 | 0.37 | 0.33 |
| Controls | Yes | Yes | Yes | Yes | Yes | Yes | Yes | Yes | Yes | Yes | Yes |

Robust standard errors reported. *** p<0.01, ** p<0.05, * p<0.1

**Table 9.2** Subgroup Analysis of Grocery Proportion (Shaanxi)

|  | No  income | Unfixed  income | Lower Income | Days | Last pay | Debt | Gender | Age | Education | Household number | Health exam |
| --- | --- | --- | --- | --- | --- | --- | --- | --- | --- | --- | --- |
|  | b/se/p | b/se/p | b/se/p | b/se/p | b/se/p | b/se/p | b/se/p | b/se/p | b/se/p | b/se/p | b/se/p |
| Group | 0.01 | -0.03 | -0.00 | 0.00 | -0.01 | -0.08 | -0.03 | -0.02 | 0.37 | -0.04 | -0.01 |
|  | 0.11 | 0.04 | 0.04 | 0.03 | 0.04 | 0.05 | 0.04 | 0.04 | 0.20 | 0.05 | 0.05 |
|  | 0.91 | 0.36 | 0.99 | 0.92 | 0.80 | 0.16 | 0.46 | 0.63 | 0.16 | 0.44 | 0.90 |
| _cons | 0.99 | 0.62 | 0.74 | 0.65 | 0.62 | 0.88 | 0.85 | 0.80 | 1.76 | 0.83 | 0.76 |
|  | 0.29 | 0.15 | 0.14 | 0.11 | 0.17 | 0.19 | 0.13 | 0.12 | 0.44 | 0.15 | 0.13 |
|  | 0.00 | 0.00 | 0.00 | 0.00 | 0.00 | 0.00 | 0.00 | 0.00 | 0.03 | 0.00 | 0.00 |
| Observations | 41 | 181 | 186 | 204 | 145 | 85 | 130 | 142 | 24 | 129 | 154 |
| R-squared | 0.40 | 0.16 | 0.17 | 0.14 | 0.16 | 0.33 | 0.29 | 0.14 | 0.93 | 0.19 | 0.17 |
| Controls | Yes | Yes | Yes | Yes | Yes | Yes | Yes | Yes | Yes | Yes | Yes |

Robust standard errors reported. *** p<0.01, ** p<0.05, * p<0.1

**Table 9.3** Subgroup Analysis of Health Proportion (Shaanxi)

|  | No  income | Unfixed  income | Lower Income | Days | Last pay | Debt | Gender | Age | Education | Household number | Health exam |
| --- | --- | --- | --- | --- | --- | --- | --- | --- | --- | --- | --- |
|  | b/se/p | b/se/p | b/se/p | b/se/p | b/se/p | b/se/p | b/se/p | b/se/p | b/se/p | b/se/p | b/se/p |
| Group | -0.03 | -0.02 | -0.03 | -0.03 | -0.04 | 0.03 | -0.02 | -0.01 | -0.19 | -0.02 | -0.04 |
|  | 0.08 | 0.03 | 0.02 | 0.02 | 0.03 | 0.04 | 0.03 | 0.03 | 0.09 | 0.03 | 0.03 |
|  | 0.72 | 0.38 | 0.24 | 0.18 | 0.11 | 0.45 | 0.42 | 0.80 | 0.12 | 0.50 | 0.24 |
| _cons | -0.13 | 0.36 | 0.19 | 0.22 | 0.23 | 0.06 | 0.10 | 0.12 | -0.70 | 0.11 | 0.25 |
|  | 0.24 | 0.11 | 0.09 | 0.07 | 0.10 | 0.13 | 0.08 | 0.09 | 0.47 | 0.10 | 0.09 |
|  | 0.60 | 0.00 | 0.04 | 0.00 | 0.03 | 0.64 | 0.24 | 0.16 | 0.24 | 0.25 | 0.01 |
| Observations | 41 | 181 | 186 | 204 | 145 | 85 | 130 | 142 | 24 | 129 | 154 |
| R-squared | 0.43 | 0.17 | 0.16 | 0.15 | 0.18 | 0.29 | 0.20 | 0.09 | 0.83 | 0.16 | 0.17 |
| Controls | Yes | Yes | Yes | Yes | Yes | Yes | Yes | Yes | Yes | Yes | Yes |

Robust standard errors reported. *** p<0.01, ** p<0.05, * p<0.1

**Table 9.4** Subgroup Analysis of Temptation Proportion (Shaanxi)

|  | No  income | Unfixed  income | Lower Income | Days | Last pay | Debt | Gender | Age | Education | Household number | Health exam |
| --- | --- | --- | --- | --- | --- | --- | --- | --- | --- | --- | --- |
|  | b/se/p | b/se/p | b/se/p | b/se/p | b/se/p | b/se/p | b/se/p | b/se/p | b/se/p | b/se/p | b/se/p |
| Group | 0.02 | 0.06 | 0.03 | 0.03 | 0.06 | 0.05 | 0.05 | 0.03 | -0.17 | 0.06 | 0.04 |
|  | 0.06 | 0.02 | 0.03 | 0.02 | 0.03 | 0.04 | 0.03 | 0.03 | 0.14 | 0.03 | 0.03 |
|  | 0.80 | 0.02 | 0.26 | 0.21 | 0.05 | 0.21 | 0.11 | 0.33 | 0.30 | 0.07 | 0.14 |
| _cons | 0.14 | 0.02 | 0.07 | 0.13 | 0.16 | 0.06 | 0.06 | 0.08 | -0.07 | 0.06 | -0.02 |
|  | 0.17 | 0.10 | 0.11 | 0.08 | 0.13 | 0.13 | 0.11 | 0.08 | 0.44 | 0.12 | 0.10 |
|  | 0.43 | 0.84 | 0.53 | 0.14 | 0.24 | 0.65 | 0.59 | 0.32 | 0.89 | 0.64 | 0.86 |
| Observations | 41 | 181 | 186 | 204 | 145 | 85 | 130 | 142 | 24 | 129 | 154 |
| R-squared | 0.54 | 0.18 | 0.15 | 0.13 | 0.18 | 0.31 | 0.23 | 0.20 | 0.87 | 0.22 | 0.15 |
| Controls | Yes | Yes | Yes | Yes | Yes | Yes | Yes | Yes | Yes | Yes | Yes |

Robust standard errors reported. *** p<0.01, ** p<0.05, * p<0.1

**Section 2. Materials and Methods**

Experimental Materials and Procedures

Experimental Materials:

Laptops: 4 units

Smartphones and power banks: 4 each (to connect to mobile hotspots for questionnaire submission to the online survey system)

Printed questionnaires: 100 copies for each, 200 in total

Coins: 4

Token gifts: 240 units

Procedures

| Step | Name | Key points |
| --- | --- | --- |
| Step 1 | Randomisation | 1) Randomly select subjects to answer Questionnaire One or Two.  2) Make sure to check the informed consent form. |
| Step 2 | Task 1: Financial scenarios | 1) Subjects read the situational questions themselves or the research assistant reads them aloud.  2) Subjects answer questions; multiple-choice questions are mandatory, and open-ended questions should be guided for imagination and description. |
|  | Task 2: Lottery game | 1) Research assistant explains the game rules; subjects choose freely according to preference.  2) Toss the coin and select the corresponding amount. |
|  | Task 3: Reward choice | Subjects choose freely according to preference. |
|  | Task 4: Budget Allocation | Subjects choose freely according to preference; the total amount should be close to 100 yuan. |
|  | Task 5: Raven's Matrices Test | Remind subjects not to randomly select or skip questions. |
|  | Task 6: Demographics | Amount and timing of income can be approximate numbers and dates. |
| Step 3 | Distribution of thankyou gifts |  |
| Step 4 | Questionnaire Entry | If using paper questionnaires, please enter the responses into the online survey system later. |

Questionnaire

1. Financial Scenarios

Instructions: In the following section you will be presented 3 scenarios and asked to answer how you would go about dealing with the situations if they were to happen to you. Please take your time answering the questions. Try to have at least 3 sentences in your open question answers.

1.1 Imagine that an unforeseen event requires of you an immediate (¥20000/¥1000) expense. You need to raise the money in less than a week.

• Are there ways in which you may be able to come up with that amount of money on a very short notice? (yes/no)

• How would you go about getting (¥20000/¥1000) on a very short notice? Three sentences should be enough. (open)

• To what extent do you agree with the following statements? (5 item Likert: strongly disagree - strongly agree)

(a) "Coming up with (¥20000/¥1000) on a very short notice would cause me long-lasting ﬁnancial hardship.”

(b) "Coming up with (¥20000/¥1000) on a very short notice would require me to make sacriﬁces that have long-term consequences.”

1.2 Imagine that the economy is going through diﬃcult times. Your household ’s income decreases by (50%/5%).

• Please indicate to what extent do you agree with the following statement: "Given my situation, I would be able to maintain roughly the same lifestyle under those new circumstances.” (5 item Likert: strongly disagree - strongly agree)

• In what ways would the (50%/5%) decrease in your income would impact your daily life? What changes would you need to make? Three sentences should be enough. (open)

• To what extent do you agree with the following statement: "The (50%/5%) decrease in our income would strongly impact our daily life." (5 item Likert: strongly disagree - strongly agree)

1.3 Imaging that due to serve illness, there is an increase in the monthly cost of healthcare by (¥2000/¥100) for your family, which amounts to a total cost increase of (¥24000/¥1200) a year. This increase is not reimbursable by any government funding scheme.

• Would it be diﬃcult to aﬀord healthcare? (yes/no)

• How would you go about covering the cost of healthcare? Would you need to make any sacriﬁces and budget cuts every month? Three sentences should be enough. (open)

• To what extent do you agree with the following statements? (5 item Likert: strongly disagree - strongly agree)

(a) "Paying additional (¥2000/¥100) a month for healthcare would require diﬃcult budget cuts and sacriﬁces every month.”

(b) "Paying additional (¥2000/¥100) a month for healthcare would be too costly and it would probably result in forgoing going to the hospital.”

2. Risk choice scenarios

Scenario 1: The following are 6 groups of lottery games. Each group of lottery contains 2 winning amounts, and the odds of winning are 50%.

Please choose which group of lottery games you prefer to play according to your own preferences:

○ lottery 1: 50% chance winning ¥28，50% chance winning ¥28

○ lottery 2: 50% chance winning ¥36，50% chance winning ¥24

○ lottery 3: 50% chance winning ¥44，50% chance winning ¥20

○ lottery 4: 50% chance winning ¥52，50% chance winning ¥16

○ lottery 5: 50% chance winning ¥60，50% chance winning ¥12

○ lottery 6: 50% chance winning ¥70，50% chance winning ¥2

Scenario 2: Imagine that there is an outbreak of an unusual disease. There are currently 6 vaccine products to choose from. Each vaccine will produce 2 possible immunity durations after vaccination, and the probability of occurrence is 50%.

Please select which vaccine you would prefer:

○ vaccine 1：50% chance immunity for 6 months，50% chance immunity for 6 months

○ vaccine 2：50% chance immunity for 8 months，50% chance immunity for 5 months

○ vaccine 3：50% chance immunity for 10 months，50% chance immunity for 4 months

○ vaccine 4：50% chance immunity for 12 months，50% chance immunity for 3 months

○ vaccine 5：50% chance immunity for 14 months，50% chance immunity for 2 months

○ vaccine 6：50% chance immunity for 17 months，50% chance immunity for 10 days

3. Time choice scenarios

Scenario 1: Imagine that after completing a job, you have two ways of getting paid:
A. to receive a smaller amount of payment earlier
B. to receive a larger amount of payment later

In the following two sets of decisions, please make the choice in which of the two ways you would like to receive the payment:

| First set (payment today vs. 1 month) | A | B |
| --- | --- | --- |
| Decision 1: A. ¥295 today B. ¥300 in 1 month | ○ | ○ |
| Decision 2: A. ¥290 today B. ¥300 in 1 month | ○ | ○ |
| Decision 3: A. ¥275 today B. ¥300 in 1 month | ○ | ○ |
| Decision 4: A. ¥265 today B. ¥300 in 1 month | ○ | ○ |
| Decision 5: A. ¥245 today B. ¥300 in 1 month | ○ | ○ |
| Decision 6: A. ¥230 today B. ¥300 in 1 month | ○ | ○ |
| Decision 7: A. ¥200 today B. ¥300 in 1 month | ○ | ○ |
| Decision 8: A. ¥180 today B. ¥300 in 1 month | ○ | ○ |

| Second set (payment in 6 months vs. 7 months) | A | B |
| --- | --- | --- |
| Decision 1: A. ¥295 in 6 months B. ¥300 in 7 months | ○ | ○ |
| Decision 2: A. ¥290 in 6 months B. ¥300 in 7 months | ○ | ○ |
| Decision 3: A. ¥275 in 6 months B. ¥300 in 7 months | ○ | ○ |
| Decision 4: A. ¥265 in 6 months B. ¥300 in 7 months | ○ | ○ |
| Decision 5: A. ¥245 in 6 months B. ¥300 in 7 months | ○ | ○ |
| Decision 6: A. ¥230 in 6 months B. ¥300 in 7 months | ○ | ○ |
| Decision 7: A. ¥200 in 6 months B. ¥300 in 7 months | ○ | ○ |
| Decision 8: A. ¥180 in 6 months B. ¥300 in 7 months | ○ | ○ |

Scenario 2: A physical examination bundle costs around 500 yuan. Suppose there is a subsidy policy, and you have two ways of receiving the subsidy:

A. to receive a smaller amount of subsidy earlier
B. to receive a larger amount of subsidy later

In the following two sets of decisions, please make the choice in which of the two ways you would like to receive the subsidy:

| First set (subsidy today vs. 1 month) | A | B |
| --- | --- | --- |
| Decision 1: A. physical exam today, subsidizing ¥295  B. physical exam in 1 month, subsidizing ¥300 | ○ | ○ |
| Decision 2: A. physical exam today, subsidizing ¥290  B. physical exam in 1 month, subsidizing ¥300 | ○ | ○ |
| Decision 3: A. physical exam today, subsidizing ¥275  B. physical exam in 1 month, subsidizing ¥300 | ○ | ○ |
| Decision 4: A. physical exam today, subsidizing ¥265  B. physical exam in 1 month, subsidizing ¥300 | ○ | ○ |
| Decision 5: A. physical exam today, subsidizing ¥245  B. physical exam in 1 month, subsidizing ¥300 | ○ | ○ |
| Decision 6: A. physical exam today, subsidizing ¥230  B. physical exam in 1 month, subsidizing ¥300 | ○ | ○ |
| Decision 7: A. physical exam today, subsidizing ¥200  B. physical exam in 1 month, subsidizing ¥300 | ○ | ○ |
| Decision 8: A. physical exam today, subsidizing ¥180  B. physical exam in 1 month, subsidizing ¥300 | ○ | ○ |

| Second set (subsidy in 6 months vs. 7 months) | A | B |
| --- | --- | --- |
| Decision 1: A. physical exam in 6 months, subsidizing ¥295  B. physical exam in 7 months, subsidizing ¥300 | ○ | ○ |
| Decision 2: A. physical exam in 6 months, subsidizing ¥290  B. physical exam in 7 months, subsidizing ¥300 | ○ | ○ |
| Decision 3: A. physical exam in 6 months, subsidizing ¥275  B. physical exam in 7 months, subsidizing ¥300 | ○ | ○ |
| Decision 4: A. physical exam in 6 months, subsidizing ¥265  B. physical exam in 7 months, subsidizing ¥300 | ○ | ○ |
| Decision 5: A. physical exam in 6 months, subsidizing ¥245  B. physical exam in 7 months, subsidizing ¥300 | ○ | ○ |
| Decision 6: A. physical exam in 6 months, subsidizing ¥230  B. physical exam in 7 months, subsidizing ¥300 | ○ | ○ |
| Decision 7: A. physical exam in 6 months, subsidizing ¥200  B. physical exam in 7 months, subsidizing ¥300 | ○ | ○ |
| Decision 8: A. physical exam in 6 months, subsidizing ¥180  B. physical exam in 7 months, subsidizing ¥300 | ○ | ○ |

4. Budget allocation

Scenario: In the following task you are asked to choose what goods to purchase with a budget of ¥100. You will see a list of available goods, with a picture, title and the price displayed for each of them. By clicking on the ‘+’ button, the goods will be added to the shopping cart. Try to spend as close to the ¥100 budget as possible.

□ rice __¥19.8__	□ Weitai granule ___¥21.6__	□ Coffee pot __¥59_____

□ Chinese leaf _¥11.3_ □ Lianhua Qingwen capsules _¥21.6_ □ Bluetooth soundbox __¥49__

□ Strawberry __¥23.5__	□ Vitamin C __¥19.8__	□ Fresh flowers __¥19.8__

□ Chicken __¥36.8_____	□ Ibuprofen capsules __¥28_	□ Calendar __¥68___

□ Milk __¥19.9__	□ Chinese herb __¥13.3__	□ Candy __¥19.9__

□ Sunflower oil __¥69__	□ Fish oil __¥58__	□ CocaCola __¥25__

5. Please select the appropriate pattern to fill in the vacancy according to the rules of the symbols or patterns in the big picture, and select the numbering option corresponding to the pattern.

5.1

 

| ○1 | ○2 | ○3 |
| --- | --- | --- |
| ○4 | ○5 | ○6 |

5.2

 

| ○1 | ○2 | ○3 |
| --- | --- | --- |
| ○4 | ○5 | ○6 |

5.3

 

| ○1 | ○2 | ○3 |
| --- | --- | --- |
| ○4 | ○5 | ○6 |

5.4

 

| ○1 | ○2 | ○3 |
| --- | --- | --- |
| ○4 | ○5 | ○6 |

5.5

 

| ○1 | ○2 | ○3 |
| --- | --- | --- |
| ○4 | ○5 | ○6 |

5.6

 

| ○1 | ○2 | ○3 |
| --- | --- | --- |
| ○4 | ○5 | ○6 |

5.7

| ○1 | ○2 | ○3 |
| --- | --- | --- |
| ○4 | ○5 | ○6 |

5.8

| ○1 | ○2 | ○3 |
| --- | --- | --- |
| ○4 | ○5 | ○6 |

5.9

 

| ○1 | ○2 | ○3 |
| --- | --- | --- |
| ○4 | ○5 | ○6 |

5.10

| ○1 | ○2 | ○3 |
| --- | --- | --- |
| ○4 | ○5 | ○6 |

5.11

| ○1 | ○2 | ○3 |
| --- | --- | --- |
| ○4 | ○5 | ○6 |

5.12

| ○1 | ○2 | ○3 |
| --- | --- | --- |
| ○4 | ○5 | ○6 |

6. Demographic information

6.1 Sex:

| ○ Male | ○ Female |  |  |  |  |  |  |
| --- | --- | --- | --- | --- | --- | --- | --- |

6.2 Age:

○ 18 years below

○ 18~25 years old

○ 26~30 years old

○ 31~40 years old

○ 41~50 years old

○ 51~60 years old

○ 60 years above

6.3 Education:

○ Illiterate

○ Primary school

○ Middle school

○ High school

○ Undergraduate and above

6.4 Annual income in 2021 (estimation):

_________________________________

6.5 Income sources:

○ Migrant Works

○ Farming

○ Family support

○ No income

○ other

6.6 Income frequency:

○ Monthly

○ Quarterly

○ Semiannually

○ Annually

○ Not fixed

6.7 Date of last payment:

_________________________________

6.8 Date of last payment:

_________________________________

6.9 Physical examination in the last 12 months:

| ○ Yes | ○ No |  |  |  |  |  |  |
| --- | --- | --- | --- | --- | --- | --- | --- |

6.10 Household size:

_________________________________

6.11 Household annual income in 2021 (estimation):

_________________________________

6.12 Household debt larger than half of household annual income:

| ○ Yes | ○ No |  |  |  |  |  |  |
| --- | --- | --- | --- | --- | --- | --- | --- |

6.13 Household healthcare expenditure larger than half of household annual income:

| ○ Yes | ○ No |  |  |  |  |  |  |
| --- | --- | --- | --- | --- | --- | --- | --- |
